# The histone demethylase KDM5C functions as a tumor suppressor in AML by repression of bivalently marked immature genes

**DOI:** 10.1038/s41375-023-01810-6

**Published:** 2023-01-12

**Authors:** Mette Louise Trempenau, Mikkel Bruhn Schuster, Sachin Pundhir, Mafalda Araujo Pereira, Adrija Kalvisa, Marta Tapia, Jinyu Su, Ying Ge, Bauke de Boer, Alexander Balhuizen, Frederik Otzen Bagger, Pavel Shliaha, Patrycja Sroczynska, Julian Walfridsson, Kirsten Grønbæk, Kim Theilgaard-Mönch, Ole N. Jensen, Kristian Helin, Bo T. Porse

**Affiliations:** 1 The Finsen Laboratory, Copenhagen University Hospital—Rigshospitalet, Copenhagen, Denmark; 2 Biotech Research and Innovation Centre, University of Copenhagen, Copenhagen, Denmark; 3 Novo Nordisk Foundation Center for Stem Cell Biology, DanStem, Faculty of Health Sciences, Faculty of Health and Medical Sciences, University of Copenhagen, Copenhagen, Denmark; 4 The Bioinformatics Centre, Department of Biology, Faculty of Natural Sciences, University of Copenhagen, Copenhagen, Denmark; 5 Department of Biochemistry and Molecular Biology and VILLUM Center for Bioanalytical Sciences, University of Southern Denmark (Odense), Odense, Denmark; 6 Department of Hematology, Rigshospitalet, Copenhagen, Denmark; 7 Department of Clinical Medicine, Faculty of Health and Medical Sciences, University of Copenhagen, Copenhagen, Denmark; 8 Cell Biology Program and Center for Epigenetics, Memorial Sloan Kettering Cancer Center, New York, NY USA; 9 The Institute of Cancer Research (ICR), London, UK; 10 Present Address: Department of Medicine, Karolinska Institutet, Huddinge, Sweden

**Keywords:** Cancer models, Acute myeloid leukaemia

## Abstract

Epigenetic regulators are frequently mutated in hematological malignancies including acute myeloid leukemia (AML). Thus, the identification and characterization of novel epigenetic drivers affecting AML biology holds potential to improve our basic understanding of AML and to uncover novel options for therapeutic intervention. To identify novel tumor suppressive epigenetic regulators in AML, we performed an in vivo short hairpin RNA (shRNA) screen in the context of *CEBPA* mutant AML. This identified the Histone 3 Lysine 4 (H3K4) demethylase KDM5C as a tumor suppressor, and we show that reduced *Kdm5c/KDM5C* expression results in accelerated growth both in human and murine AML cell lines, as well as in vivo in *Cebpa* mutant and inv(16) AML mouse models. Mechanistically, we show that KDM5C act as a transcriptional repressor through its demethylase activity at promoters. Specifically, KDM5C knockdown results in globally increased H3K4me3 levels associated with up-regulation of bivalently marked immature genes. This is accompanied by a de-differentiation phenotype that could be reversed by modulating levels of several direct and indirect downstream mediators. Finally, the association of *KDM5C* levels with long-term disease-free survival of female AML patients emphasizes the clinical relevance of our findings and identifies *KDM5C* as a novel female-biased tumor suppressor in AML.

## Introduction

AML is an aggressive blood cancer characterized by rapid accumulation of immature myeloid precursors in bone marrow (BM) and peripheral organs. Patient survival remains poor for most AML subtypes, thus a deeper understanding of AML biology is needed. This is highlighted by the success of a few targeted treatments for specific AML subtypes including all-trans retinoic acid for treatment of t(8;21) AML [1].

Epigenetic factors along with transcription factors, growth regulators and splicing factors are frequently mutated in AML, and their perturbation in pre-malignant cells can provide a permissive environment for transformation and tumorigenesis [2]. Epigenetics typically defines the reversible chemical modifications to DNA and histones regulating chromatin accessibility and transcription without changing the DNA sequence. For example, the presence of H3K4me3 (activating) and H3K27me3 (repressive) histone modifications at the same promoter maintains a bivalent “ready-to-fire” state often seen at promoters of lineage-specific genes in embryonic stem cells (ESCs) and hematopoietic stem cells (HSCs) [3, 4].

The coordinated actions of epigenetic readers, writers, and erasers are important for transcriptional regulation and cell identity [5]. Consistently, epigenetic dysregulation is frequent in AML, and factors involved in DNA methylation and histone modification are recurrently mutated in AML patients [6] or dysregulated transcriptionally [7]. Here we assessed the importance of epigenetic dysregulation in AML by conducting an in vivo shRNA screen in a mouse model of bi-allelic *CEBPA* mutant AML. Specifically, AML development in this model is driven by the homozygous expression of the *Cebpa*^*Lp30*^ allele thereby phenocopying bi-allelic *CEBPA* mutant AML (affecting 7–10% of AML patients) where the truncated CEBPA-p30 variant constitutes the sole functional CEBPA isoform [8, 9]. As *CEBPA* mutant AML is associated with favorable prognosis [10], this makes the Lp30 model especially suitable for assessing an accelerated disease latency phenotype. Using this set-up, we identified KDM5C, a H3K4me2/me3 demethylase, as a tumor suppressor in AML and further validated its function in cell lines and primary human AML. We found that *Kdm5c* knockdown (KD) leads to transcription from low-activity and bivalent/repressed promoters associated with high KDM5C binding causing upregulation of genes normally expressed in immature progenitors, resulting in a more aggressive leukemia. Furthermore, we found that *KDM5C* levels predicts outcome in female AML patients thus identifying KDM5C as a female-biased tumor suppressor in AML.

## Methods

### Animal experiments

C57BL6/6 J.SJL congenic recipients (female, 10–15 weeks old) were sub-lethally irradiated (500 cGy) 8–18 h prior to intravenous injection with *Cebpa*^*Lp30/Lp30*^ (from hereon, Lp30) AML cells [8]. When indicated, cells were premixed with 4.5 × 10^5^ irradiated (2000 cGy) non-engrafting carrier cells per recipient.

See supplementary methods for detailed descriptions.

### Plasmids

Plasmids and cloning procedures are described in Supplementary Methods.

### In vitro experiments

All in vitro experiments are described in detail in Supplementary Methods.

### Flow cytometry analysis

Femur, tibia, and hip bones were harvested from pre-leukemic mice, crushed, using mortar and pestle, and resuspended in phosphate buffed saline (PBS) with 3% fetal bovine serum (FBS). Flow cytometry was performed as described in Supplementary methods using antibodies list in Supplementary Table 2.

### RNA preparation and RT-qPCR

RNA was extracted from 1–5 × 10^5^ cells using the RNeasy Kit (Qiagen) according to the manufacturer’s recommendation. cDNA was produced using the Protoscript M-Mulv First Strand Synthesis Kit (New England Biolabs). Primers (Supplementary Table 3) for RT-qPCR were designed using Primer3 [11] or Primer-Blast [12] software with an optimal annealing temperature of 60 °C.

### Chromatin immunoprecipitation sequencing (ChIP-seq)

ChIP-seq was performed on 5 × 10^5^ FACS-sorted GFP^+^ shControl and sh*Kdm5c*-I Lp30 cells from murine BM as previously described [13].

Antibodies: a-H3K4me3 (9751 S, Cell Signaling), a-H3K4me1 (ab8895, Abcam), a-H3K27ac (ab4729, Abcam), a-H3K27me3 (C36B11, Cell Signaling) and IgG (I8140, Sigma).

ChIP-conditions and bioinformatical analyses are described in detail in Supplementary methods.

### Data access

Sequencing data can be retrieved from Gene Expression Omnibus (GSE141477). The mass spectrometry data are retrievable from the ProteomeXchange Consortium via the PRIDE partner repository (PXD016568). The shRNA screen data are provided in Supplementary Tables 4–7. All other data are available from the authors on request.

### Data analysis, visualization, and statistics

All graphs and analysis were created using GraphPad Prism 7.0 or R [14]. Illustrations were made using Adobe Illustrator CS6 version 16.0.0.

Data were analyzed for significance using One-Way-Anova for multiple comparisons and *T*-test for pairwise comparisons. Error bars indicate standard deviations unless otherwise stated. For Kaplan–Meier plots, *p*-values were calculated using a Log-rank test. *P* < 0.05 were considered significant. In vivo experiments were carried out in biological replicates, whereas in vitro experiments were carried out in technical replicates. Unless otherwise stated, experiments were carried out once. No blinding of experimental groups was performed. No statistical method was applied to predetermine sample sizes, but sample sizes are indicated in relevant figures. For BMT experiments, recipient mice were randomized to receive control and test leukemic cells, respectively. For survival analysis, animals were shuffled between cages.

## Results

### Identification of *Kdm5c* as a putative tumor suppressor in AML

**Fig. 1:**
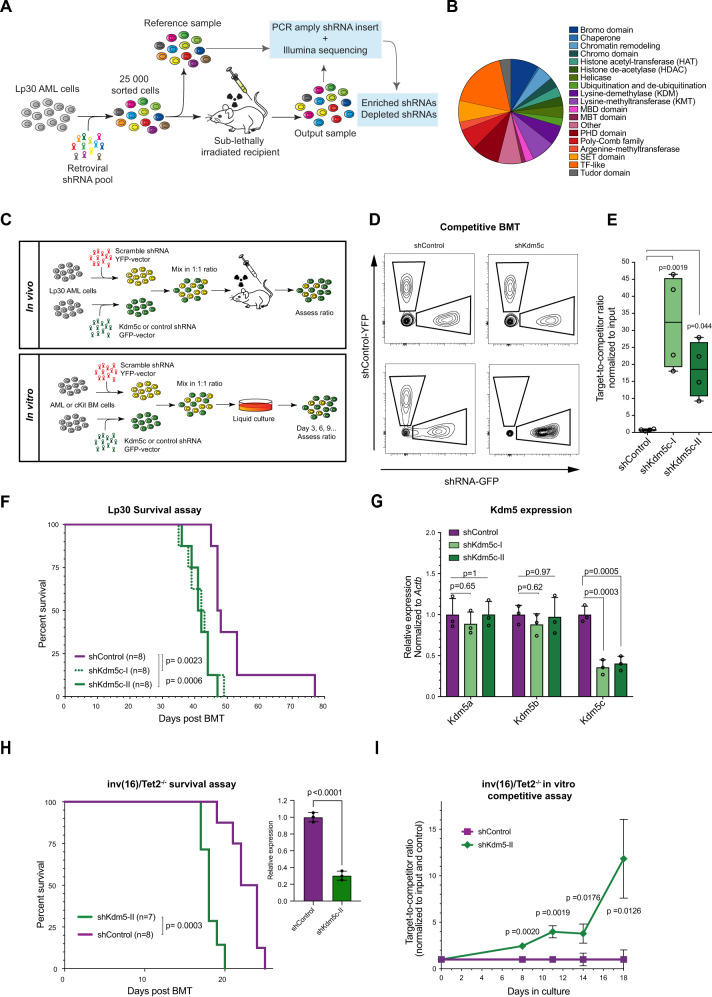
*Kdm5c*-knockdown is associated with a competitive advantage and decreased latency in AML. **A** Schematic outline of the in vivo shRNA screen targeting chromatin associated factors. **B** Relative proportions of chromatin-associated factors targeted in the shRNA-library based on Gene Ontology analysis. The library consists of 849 shRNAs targeting 315 genes. **C** Schematic outline of the competitive assay in vivo (top panel) and in vitro (bottom panel). The in vivo competitive assay was performed by BMT of Lp30 cells transduced with target-shRNA (GFP-selection) or competitive non-targeting shRNA (YFP-selection) in a 1:1 GFP/YFP ratio. The competitive advantage was assayed by flow cytometry of the BM 3 weeks later. For the in vitro competitive assay, a fraction of the cell culture was analyzed by flow cytometry at each passage. **D** Representative flow cytometry profiles of input and output (4 weeks post-BMT) of shControl and *Kdm5c*-KD groups. Target-shRNA-transduced cells are GFP-positive versus YFP-positive BM cells transduced with competitive non-targeting control-shRNA. **E** Performance of Lp30 *Kdm5c*-KD cells in BMT normalized to input ratio of target/competitor cells (*n* = 4 per group). **F** Survival analysis of mice with transplanted shControl or *Kdm5c*-KD Lp30 cells (*n* = 8 per group). **G** Relative expression of *Kdm5*a, *Kdm5b,* and *Kdm5*c in shControl and *Kdm5c*-KD groups assayed by RT-qPCR and normalized to *Actb* expression. **H** Survival analysis of mice transplanted with Inv(16)/*Tet2*^*−/−*^ cells transduced with shControl or sh*Kdm5c*. Insert: Relative expression of *Kdm5a* in shControl and *Kdm5c*-KD groups assayed by RT-qPCR and normalized to Actb expression. Relative expression of *Kdm5*c in shControl and *Kdm5c*-KD cells was assayed by RT-qPCR and normalized to *Actb* expression. These data are a representative example of two independent experiments. **I** Competitive culture of control and Kdm5c-KD Inv(16)/*Tet2*^*−/−*^ cells.

To identify factors affecting AML progression, we performed a pooled in vivo shRNA screen in the transplantable murine *Cebpa*^*Lp30/Lp30*^ (Lp30) AML model [8] using a library of 849 shRNAs targeting 315 chromatin-associated factors (Fig. 1A, B; Supplementary Tables 4–6) [15]. The screen methodology has previously been reported [16], and relies on the fact that targeting of oncogenes and tumor suppressors will lead to depletion and selection, respectively, of affected clones in a pooled setting. Briefly, Lp30 murine AML cells transduced with pools of shRNA-encoding retrovirus were transplanted to sub-lethally irradiated recipient mice. BM cells from these recipients were harvested after four weeks, and the shRNA repertoires at this timepoint were compared to input cells to identify shRNAs depleted or enriched during in vivo growth of the tumor.

Here, we focused on enriched shRNAs targeting candidate tumor suppressors. shRNAs were ranked by their mean fold-change (FC), and genes were scored as hits if targeted by multiple shRNAs ranking within the top 25th percentile of enriched shRNAs. The list of potential tumor suppressor hits included both known factors, such as *Ezh2* and *Tle4*, as well as novel candidates, including *Kdm5c*, *Pcgf3, Chd1* and *Pbrm1* (Fig. S1A–C). KDM5C belongs to a family of four histone demethylases, KDM5A-D, which are ubiquitously expressed in the hematopoietic system (Fig. S1D). It catalyzes the demethylation of di- and tri-methylated Lysine 4 on Histone 3 (H3K4me2/3) [17, 18] and was the only KDM5 member scoring in our screen (Fig. S1A; Supplementary Table 7). All KDM5 members were minimally deregulated at the protein level in Lp30 AML versus normal progenitors (Fig. S1E). *Kdm5c* is found mutated in neurological disorders [19, 20] and in various cancer types, including clear cell renal cell carcinoma (ccRCC) [21–23], breast cancer [24], and AML [25–27]. Similarly, *KDM5C* scored in a CRISPR-screen in human AML cell lines [28] but the functional implications of its dysregulation in AML has not been described. We, therefore, forwarded *Kdm5c* for further functional analysis. *Kdm5c/KDM5C* is X-linked and escapes X-inactivation in female cells [29]. Conversely, male cells express the highly redundant Y-linked *Kdm5d/KDM5D* locus. Since this could potentially result in confounding compensatory effects in male cells, we chose to exclusively focus on female cell lines and animal models (including Lp30 cells) for functional experiments.

### *Kdm5c*-knockdown is associated with a competitive advantage in murine AML

We first validated the impact of *Kdm5c*-KD in Lp30 AML by competitive BM transplantation of 1:1 mixtures of “target” cells (expressing either *Kdm5c*-shRNA or non-targeting shRNA (GFP^+^)) and “competitor cells” (expressing non-targeting shRNA (YFP^+^)) and subsequently analyzed the BM “target/competitor” ratio by flow cytometry (Fig. 1C, D). Supporting a tumor suppressive function, *Kdm5c*-KD resulted in a profound enrichment (19-32 fold) of cells expressing *Kdm5c-*targeting shRNAs compared to control AML cells (Fig. 1E).

Next, we assessed whether *Kdm5c*-KD affected the survival of mice transplanted with sorted GFP^+^ Lp30 AML cells. Mice transplanted with *Kdm5c-*KD cells lived significantly shorter than the control group (median survival of 41.5/42.5 days vs. 47.5 days; Fig. 1F). Importantly, *Kdm5c-*targeting shRNAs did not affect other KDM5 members (Fig. 1G and S1F) thereby supporting a specific role for KDM5C in AML.

To investigate whether this phenotype was unique for *CEBPA* mutant AML, we performed a series of in vivo and in vitro experiments using two additional AML models. Firstly, we performed a survival experiment in mice transplanted with Inv(16); *Tet2*^*−/−*^ AML cells [30] and, in concordance with the effect in Lp30 cells, found that *Kdm5C*-KD resulted in shorter survival times (Fig. S1G). Secondly, *Kdm5C*-KD resulted in a mild competitive advantage in vitro growth assays in both Inv(16)/*Tet2*^*−/*−^ cells and MLL-AF9-transformed cells [31] (Fig. 1H and S1H, I). Except for a mild depletion of sh*Kdm5c-*II expressing cells (potentially due to off-target effects) *Kdm5c* KD had no effects in normal c-Kit-enriched BM cells (Fig. 1I). Combined, these data demonstrate that *Kdm5c* KD results in a selective growth advantage in malignant hematopoietic cells which appears absent in their normal counterparts.

To rule out potential shRNA off-target effects, we performed CRISPR-Cas9 knockout (KO) experiments in MLL-AF9 cells (Fig. S2A). CRISPR KO efficiently reduced protein expression while moderately affecting *Kdm5c* RNA levels (Fig. S2B, C). Consistent with the shRNA-KD data, *Kdm5c*-KO resulted in faster growth (Fig. S2D, E). This was associated with a positive, albeit mild, selection for deletions (gRNA-1) and insertions (gRNA-2) in the *Kdm5c* gene over time (Fig. S2F, G).

In summary, *Kdm5c*-KD led to a competitive advantage in three different murine AML models and was associated with reduced median survival of AML in vivo. Collectively, these results confirm a tumor suppressive role for KDM5C in AML.

### *Kdm5c*-knockdown leads to increased H3K4me3 at promoters and enhancers

KDM5C has been reported to regulate H3K4-methylation at active (H3K4me3^high^, H3K27ac^high^), at primed/bivalent (H3K4me3^low^, H3K27me3) promoters and active/poised (H3K4me1, +/− H3K27ac) enhancers [17, 32, 33]. To assess regional changes in the substrate (H3K4me3) and product (H3K4me1) of KDM5C at promoter- and enhancer sites, we performed Chromatin Immuno-Precipitation sequencing (ChIP-seq) of H3K4me1, H3K4me3, H3K27me3, and H3K27ac in transplanted *Kdm5c*-KD and control Lp30 AML cells. Due to lack of ChIP-grade antibodies, we were unable to assess KDM5C-binding, and we, therefore, correlated our data with published biotin-tagged KDM5C ChIP-seq data derived from ESCs and neuronal progenitor cells (NPCs) [34].

**Fig. 2:**
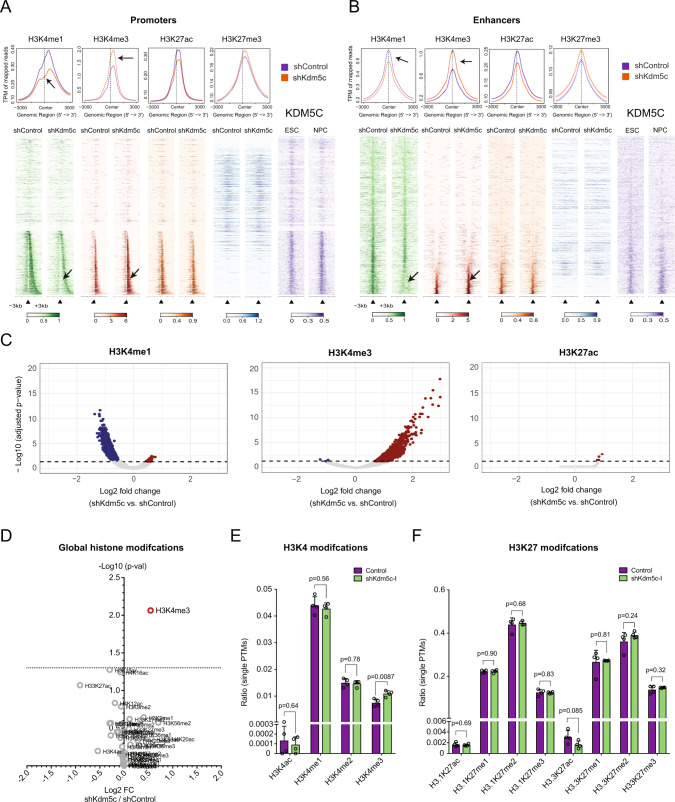
*Kdm5c*-KD leads to enrichment of H3K4me3 and displacement of H3K4me1. **A**, **B** Top panels: Average line plot of mean H3K4me1, H3K4me3, H3K27me3, and H3K27ac read density (transcript per million, TPM) at promoters (**A**) and enhancers (**B**) in control vs *Kdm5c*-knockdown (sh*Kdm5c*-I) Lp30 cells. Arrows indicate displacement of H3K4me1 and the increase of H3K4me3 levels. Lighter shaded ribbons represent standard error of mean. Bottom panel: Heatmaps of mean H3K4me1, H3K4me3, H3K27me3, and H3K27ac signal at all promoters (**A**) and enhancers (**B**) in Lp30 shControl and sh*Kdm5c*-I cells ranked based H3K4me3-peak widths (plotted 5’ to 3’ the individual TSS). Regions with no detected H3K4me3 peaks have been randomly clustered. Small arrows indicate the center of TSS or enhancer peaks. KDM5C binding in ESCs and NPCs have been included for comparison. **C** Volcano plot of differential peaks of H3K4me1 (left), H3K4me3 (center) and H3K27ac (right) in *Kdm5c*-KD (sh*Kdm5c*-I) vs control Lp30 cells. Quantified from biological duplicate ChIP-seqs. **D** Volcano plot of global differentially expressed ratios of histone-modifications in *Kdm5c*-KD (sh*Kdm5c*-I) vs control Lp30 cells determined by mass spectrometry. **E**, **F** Ratios of post-translational modifications (PTMs) at H3K4 (**D**) and K27 (**E**) of two Histone 3 variants (H3.1 and H3.3.) in *Kdm5c*-KD (sh*Kdm5c*-I) vs control Lp30 cells. *P*-values were calculated using two-tailed *T*-tests (not adjusted for multiple testing).

*Kdm5c*-KD led to an increase in H3K4me3 and a reduction of H3K4me1 at promoter and enhancer regions with expected KDM5C-binding (Fig. 2A, B and S3A–C). To further study the regional importance of KDM5C, we identified differentially marked promoter and enhancer peaks (Fig. 2C). Consistent with its enzymatic activity, *Kdm5c-*KD led to increased H3K4me3- and decreased H3K4me1-levels at thousands of promoters and enhancers, thereby demonstrating a marked epigenetic re-wiring.

To assess the effect of *Kdm5c*-KD on global histone modification levels, we performed mass spectrometry in *Kdm5c*-KD and control Lp30 AML cells (Fig. 2D–F and S3D–G; Supplementary Table 8). Consistent with our ChIP-seq data, mass spectrometry demonstrated a tendency towards a global increase in H3K4me3 levels in *Kdm5c*-KD Lp30 cells which was significant when not adjusting for multiple testing (Fig. 2D, E). Furthermore, no other histone modifications significantly changed in *Kdm5c*-KD Lp30 cells.

In summary, *Kdm5c-KD* is associated with increased H3K4me3- and decreased H3K4me1-levels, at regions with expected KDM5C binding, without major effects on additional histone modifications.

### Increased H3K4me3 levels at bivalent gene promoters are associated with a gain in gene expression

**Fig. 3:**
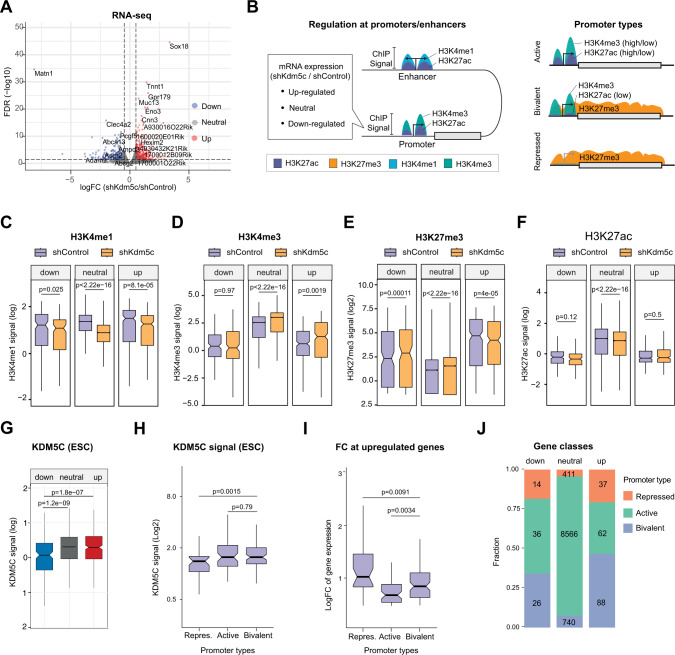
H3K4me3-gain at promoters and enhancers correlates with the upregulation of bivalent and repressed genes. **A** Volcano plot of gene expression changes in *Kdm5c*-KD vs control Lp30 cells. Quantified from RNA-seq from biological triplicates. **B** Right panel: Schematic overview of coupled analysis between RNA-seq and ChIP-seq data. Gene promoters are divided based on transcriptional regulation (up, down and neutral), and ChIP-signals at promoters or nearby enhancers are compared between sh*Kdm5c*-I and shControl cells. Left panel: Schematic overview of gene promoters and their association with different of H3-modifications (**C**–**F**) Notched boxplots showing H3K4me1- (**C**), H3K4me3- (**D**), H3K27me3- (**E**), or H3K27ac-signal (**F**) at promoters of up-, down- or neutrally regulated genes in *Kdm5c-*KD (sh*Kdm5c*-I) cells vs control. Outliers not shown. **G** Notched boxplot showing KDM5C-signal in ESCs (Outchkourov et al. 2013) [34] at promoters of up-, down- or neutrally regulated genes in sh*Kdm5c*-I vs shControl cells. Outliers not shown. **H** Notched boxplot of KDM5C-signal in ESCs (Outchkourov et al. 2013) [34] at repressed (H3K27me3), active (H3K4me3), and bivalent promoters (H3K4me3, H3K27me3) in shControl cells. Outliers not shown. **I** Notched boxplot showing expression foldchange at different promoter types (defined in **H**) of upregulated genes in sh*Kdm5c-I* vs shControl Lp30 cells. **J** Fraction of active, bivalent, and repressed promoters (defined as in **H**) at up-, down-, or neutrally regulated genes in sh*Kdm5c*-KD cells.

KDM5C has both transcriptionally activating and repressive functions and its transcriptional role may therefore be context-dependent [34, 35]. To gain further mechanistic insights into KDM5C-mediated gene regulation in AML, we performed RNA-sequencing (RNA-seq) on transplanted *Kdm5c*-KD and control Lp30 AML cells (Fig. 3A and S4A–E). Overall, we found 489 differentially expressed genes with 322 up- and 167 downregulated genes (FDR < 0.05 for either shRNA; Fig. 3A; Supplementary Table 9). Both *Kdm5c*-shRNAs resulted in comparable transcriptional deregulation (Fig. S4A–D).

To determine the effect of KDM5C activity on gene expression, we divided genes based on transcriptional deregulation (Up, Down, or Neutral; Fig. S4E) in *Kdm5c*-KD vs control and correlated gene expression with ChIP-signals of H3K4me1, H3K4me3, H3K27ac, H3K27me3, and previously published KDM5C binding data [34] at promoters/enhancers (Fig. 3B). Interestingly, H3K4me1 and H3K4me3 levels changed significantly at promoters of both neutral- and upregulated genes in *Kdm5c-KD* cells, while changes in H3K27me3-levels correlated inversely with transcriptional changes (Fig. 3C–E). In contrast, modest or no changes were observed in H3K27ac across gene classes (Fig. 3F). In concordance with the H3K4me3-changes, KDM5C occupancy was higher at promoters of neutral/upregulated genes compared to down-regulated genes (Fig. 3G and S4F). Although not a direct proof, these data suggest that transcriptional upregulation is a direct consequence of loss of a KDM5C-generated repressive promoter mark.

Of note, deregulated genes had markedly lower RNA expression and H3K4me3 levels as well as higher H3K27me3 levels compared to neutral genes in control cells (Fig. S4E, G). Thus, we hypothesized that *Kdm5c*-KD affected low-activity promoters to a higher degree. Indeed, in concordance with H3K4me3-changes, both active and bivalently marked (low-activity) promoters were associated with high KDM5C occupancy (Fig. 3H). Additionally, *Kdm5c*-KD increased expression from bivalent promoters to a larger extent than that from active promoters (Fig. 3I, J and Fig. S4H; Supplementary Table 9). Collectively this indicates that bivalent promoters are more affected by *Kdm5c*-KD than promoters with robust activity.

In contrast, down-regulated genes were associated with shorter CpG-island length at promoters and a higher number of predicted enhancer interactions compared to upregulated genes (Fig. S4I, J). We, therefore, investigated whether loss of KDM5C led to reduced enhancer-driven transcription due to reduced H3K4me1 as previously described [34]. However, we found that H3K4me1, H3K4me3, H3K27ac, and H3K27me3 levels at enhancers follow their global changes, irrespectively of expression changes of the nearest gene (Fig. S4K–N). Finally, as a proxy for reduced KDM5C activity, we assessed the distribution of enhancers with deregulated H3K4me1/3-levels but found no significant correlation to expression changes (Fig. S4O). Thus, transcriptional downregulation is likely an indirect effect of *Kdm5c*-KD.

In summary, *Kdm5c*-KD leads to de-repression of low-activity bivalent promoters associated with high KDM5C occupancy concomitantly with an increase in H3K4me3 occupancy. Thus, upregulated genes are likely a direct result of loss of KDM5C-mediated H3K4me2/3 demethylation. In contrast, we could not link transcriptional downregulation directly to KDM5C activity at enhancers.

### *Kdm5c*-KD leads to de-differentiation of Lp30 AML

**Fig. 4:**
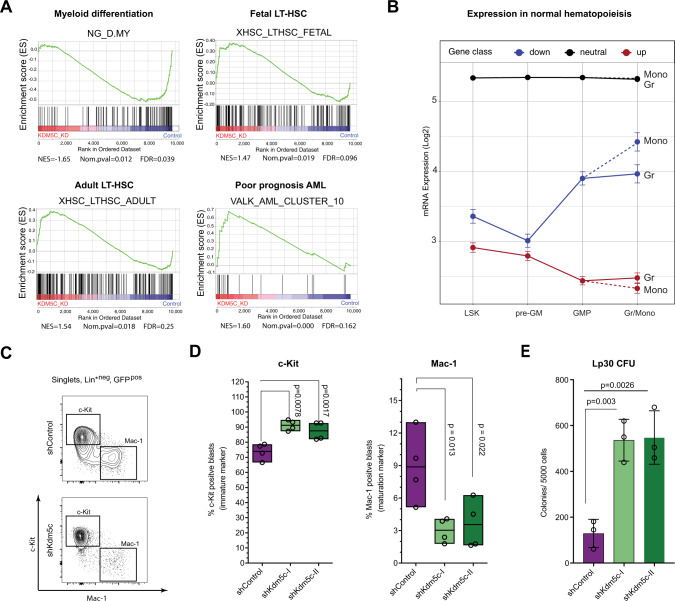
*Kdm5c*-KD leads to a de-differentiation phenotype. **A** Gene Set Enrichment Analyses showing enrichment or depletion of specific gene sets: myeloid differentiation (NG_D_MY), fetal long-term HSC (XHSC_LTHSC_FETAL), adult long-term HSC (XHSC_LTHSC_ADULT) and poor prognosis AML signature (VALK_AML_CLUSTER_10). **B** Mean Log2 expression of up-, down- and neutrally regulated genes throughout normal myeloid differentiation stages (immature to mature). LSK Lin^−^ Sca-1^+^ c-Kit^+^ cells, pre-GM pre-granulocyte/monocyte precursors, GMP Granulocyte-monocyte-progenitors, Gr granulocytes and Mono monocytes. Data derived from [54]. **C** Flow cytometry gating strategy for Lp30 AML (singlets, 7-AAD negative). **D** Frequency of control and *Kdm5c*-KD Lp30 cells with immature (c-Kit+) and mature (Mac-1+) immune-phenotype. **E** Colony-Forming-Unit (CFU) assay of Lp30 shControl and *Kdm5c-*KD cells. Quantification of dense colonies per 5000 seeded GFP + cells sorted from triplicate mice.

To explore the potential perturbation of specific molecular pathways following *Kdm5c*-KD, we performed Gene Set Enrichment Analysis (GSEA) and mainly found deregulation of differentiation-related signatures (Fig. 4A). Bivalent genes are especially enriched in immature stages and are generally resolved during differentiation [36]. We, therefore, investigated the expression of de-regulated genes through normal myelopoiesis (Fig. 4B). Indeed, upregulated genes were mainly expressed in HSC-enriched Lin^−^ Sca-1^+^ c-Kit^+^ (LSK) cells and decreased towards maturation. Oppositely, down-regulated genes were mainly expressed in more mature cells.

Consistent with this de-differentiation phenotype at the level of gene expression, *Kdm5c*-KD lead to increased frequencies of immature (c-Kit^+^) and lower frequencies of mature (Mac-1^+^) Lp30 cells (Fig. 4C, D). We observed a similar tendency in *Kdm5c-*KD MLL-AF9 cells (Fig. S5A, B). Supporting the immature phenotype, *Kdm5c*-KD Lp30 AML cells displayed significantly higher colony-forming capacity in vitro (Fig. 4E). Although *Kdm5c*-KD was not associated with any detectable changes in cell cycle or apoptosis in the total leukemic population (Fig. S5C–E), we found that the c-Kit^+^ cells cycle 2.9-fold faster than Mac-1^+^ cells (Fig. S5F, G). Higher abundance of this population is therefore likely to drive the accelerated aggressiveness of *Kdm5c*-KD AML.

To summarize, *Kdm5c*-KD primarily leads to the deregulation of differentiation-associated genes, tipping the balance towards a more immature leukemia with increased proliferative capacity.

### Direct and indirect downstream mediators facilitate the *Kdm5c*-KD phenotype

**Fig. 5:**
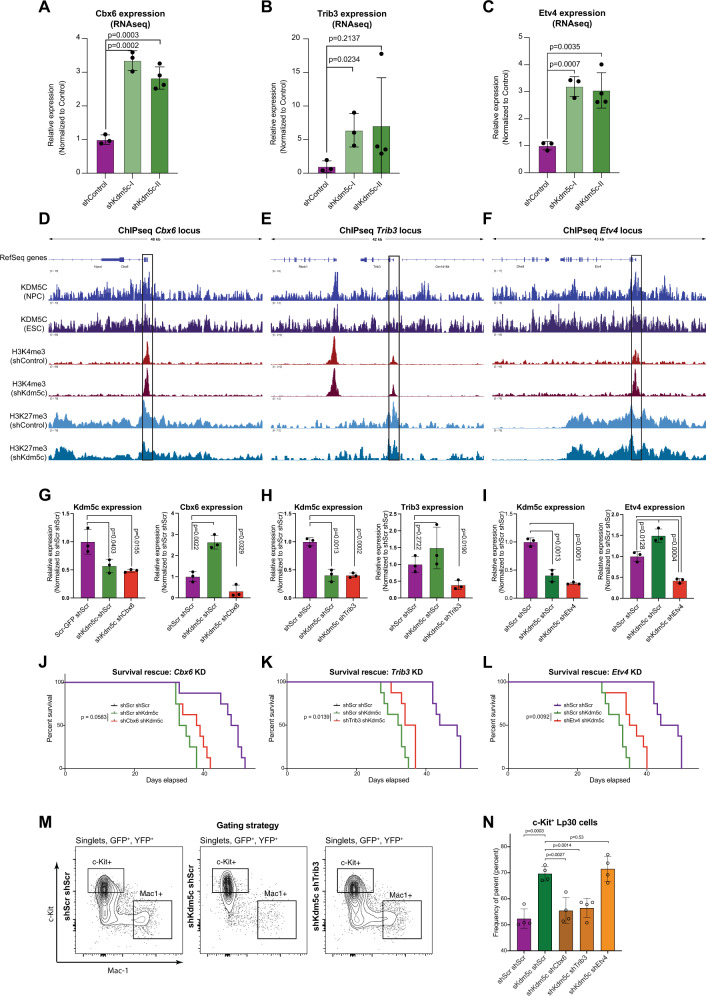
Direct and indirect downstream mediators facilitate the *Kdm5c*-KD phenotype. **A**–**C** mRNA levels of *Cbx6* (**A**), *Trib3* (**B**) and *Etv4* (**C**) (determined by RNAseq) in response to *Kdm5c*-KD **D**–**F** Occupancy of KDM5C (ESC/NPC) and changes in the indicated histone marks at the promoters of *Cbx6* (**D**), *Trib3* (**E**) and *Etv4* (**F**) (boxes) in response to *Kdm5c*-KD Lp30 cells. **G**–**I** mRNA levels of *Kdm5c* and *Cbx6* (**G**), *Trib3* (**H**) and *Etv4* (**I**) (determined by qPCR) in response to transduction with the indicated shRNAs **J**–**L** Kaplan–Meier survival analysis of mice transplanted with Lp30 cells transduced with either control or *Kdm5c*-shRNA in combination with either shScr, sh*Cbx6* (**J**), sh*Trib3* (**K**) or sh*Etv4* (**L**) (*n* = 8 per group). **M** Gating strategy for Lp30 AML (singlets, DAPI negative, GFP positive, YFP positive, assessed in BM, 3 weeks post-BMT). **N** Frequency of Lp30 cells with an immature (c-Kit^+^) immunophenotype in vivo after double transduction with the indicated shRNA constructs.

Given the effect of *Kdm5c*-KD on bivalent promoters, we wanted to assess putative functional effects of up-regulated genes on the tumor growth phenotype of the KD. To this end, we were specifically intrigued by the upregulation of the three bivalent genes Chromobox 6 (*Cbx6*), Tribbles Pseudokinase 3 (*Trib3*) and Ets Translocation Variant 4 (*Etv4*) genes, all of which were upregulated after *Kdm5c-KD* (Fig. 5A–F, Supplementary Table 9). Notably, ETV4 has numerous implications as an adverse prognostic factor in multiple cancer types [37], including ccRCC, which is prominently associated with KDM5C mutations [38].

To address the putative roles of *Cbx6*, *Trib3,* and *Etv4* as downstream effectors of the tumor promoting effect of *Kdm5c* KD, we assessed the consequences of KD of either of them in combination with *Kdm5*c KD. In accordance with the RNAseq data, qRT-PCR revealed a slight *Kdm5c* KD-mediated upregulation of each of the three genes, which was reversed by co-transduction with either of their respective shRNAs (Fig. 5G–I). Strikingly, KD of the three genes partially rescued the accelerated aggressiveness mediated by *Kdm5c* KD, further suggesting that KDM5C exerts its function via multiple downstream effectors (Fig. 5J–L).

Moreover, for *Cbx6* and *Trib3*, these effects were accompanied by rescue of the *Kdm5c* KD-dependent de-differentiation (Fig. 5M, N). Such rescue was not detected after *Etv4* KD suggesting that the impact of *Kdm5c* KD on myeloid maturation is necessary, but not sufficient to achieve the hyper-proliferative phenotype.

Among genes down-regulated following *Kdm5c-*KD, we detect *Fos* and *Jun,* both of which encode well-established myeloid transcription factors by associating with CEBPA or PU.1 to promote monocytic differentiation (Fig. S6A, B). Although we have no indication of this being a direct effect of KDM5C loss, their downregulation may still contribute to the *Kdm5c* KD phenotype. Indeed, we found that shRNA-mediated knockdown of either *Fos* or *Jun*, partially mimicked the tumor advantage of *Kdm5c*-KD in Lp30 AML (Fig. S6C–G). Conversely, over-expression of JUN extended the median survival and partially rescued *Kdm5c*-KD (Fig. S6H). Similar to *Etv4-KD* in the context of *Kdm5C*, downregulation of *Jun* expression (in a *Kdm5c-*unperturbed setting) did not impact on the differentiation status of Lp30 AML cells (Fig. S6I).

In conclusion, our data demonstrate that the impact of *Kdm5c-*KD is mediated by an ensemble of direct and indirect downstream targets, some of which affect the differentiation status of AML cells

### Low *KDM5C* expression promotes poor outcome in human AML

**Fig. 6:**
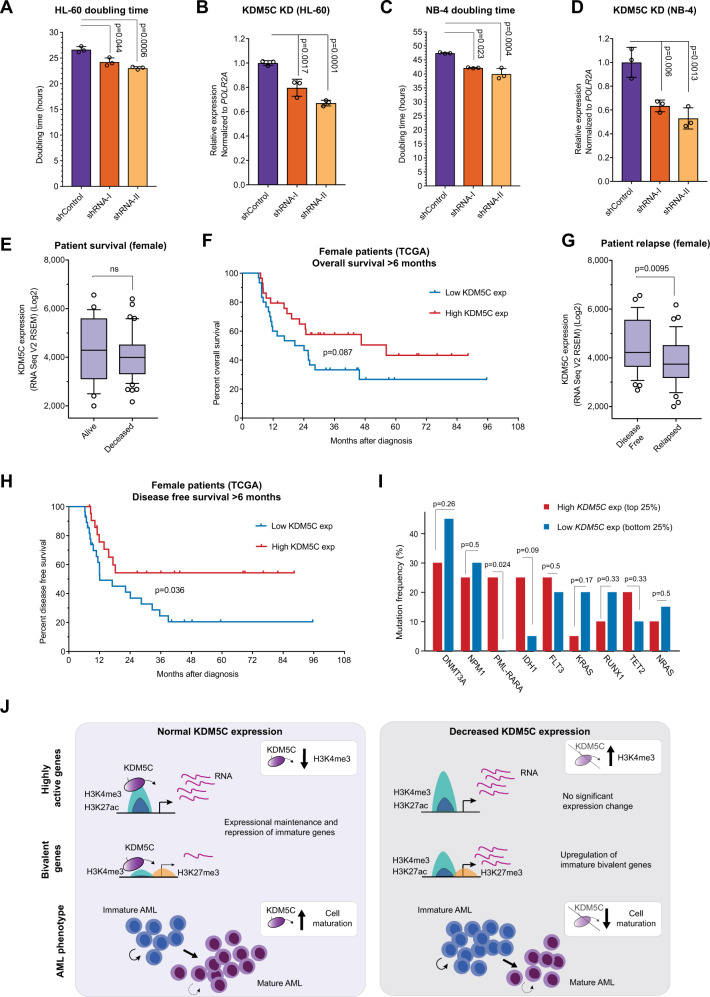
Prognostic value of KDM5C expression in human AML. **A** Doubling time (hours) of control and KDM5C-KD HL-60 cells. **B** Relative mRNA expression of *KDM5C* in HL-60 control and *KDM5C*-KD groups assayed by RT-qPCR and normalized to *POLR2A* expression. **C** Doubling time (hours) of control and *KDM5C*-KD NB-4 cells. **D** Relative mRNA expression of *KDM5C* in NB-4 control and *KDM5C*-KD groups assayed by RT-qPCR and normalized to *POLR2A* expression. **E** Correlation between *KDM5C* expression and overall survival status in female patients (TCGA dataset). P-value was calculated by unpaired two-tailed *T*-test with Welch’s correction. **F** Overall survival of female AML patients (TCGA dataset) grouped according to High (above median) and Low (below median) expression of *KDM5C* as indicated. Patients with survival times less than six months were excluded from the analysis. **G** Correlation between *KDM5C* expression and disease-free survival status in female patients (TCGA dataset). *P*-value was calculated by unpaired two-tailed *T*-test with Welch’s correction. **H** Disease-free survival of female AML patients (TCGA dataset) grouped according to High (above median) and Low (below median) expression of *KDM5C*. Patients were excluded as in (**F**). **I** Most frequent mutations found in top/bottom 25% of *KDM5C* High and Low female patients (TCGA dataset). P-values determined by Fisher’s exact test. **J** Model of KDM5C function in AML. Decreased *Kdm5c* expression leads to a general enrichment of H3K4me3 at KDM5C-targeted genes, providing leeway for increased expression from bivalently marked gene promoters (low-activity) without affecting highly active genes. These KDM5C-targeted bivalent genes are expressed in immature differentiation stages, thus their upregulation in *Kdm5c-*KD Lp30 cells results in a tumor-advantageous de-differentiation.

In hematopoietic malignancies, rare KDM5C mutations cluster in and around the catalytic Jmj-, PHD1- and Arid domains (Fig. S7A and Supplementary Table 10) and mutations in these regions have previously been associated with reduced enzymatic function or protein stability [17, 20, 39]. To functionally determine the relevance of KDM5C deregulation in human AML, we knocked down *KDM5C* in two human AML cell lines. In support of the murine phenotypes, even limited *KDM5C*-KD was associated with a 10–15% increase in growth rates in both HL-60 and NB-4 cell lines (Fig. 6A–D and S7B–D). Furthermore, we subjected primary AML cells to CRISPR-mediated targeting of the *KDM5C* and *AAVS1* loci, with the latter serving as a “safe harbor” control [40], and cultured cells in a 1:1 ratio. We could only detect insertions and/or deletions (indels) in one out of four AML samples. In this sample, the frequency of a 22 bp deletion at *KDM5C* increased from <1% to around 7% at day 16 (Fig. S7E). In contrast, *AAVS1* edits only increased from 11% to 15% indicating a specific growth advantage for *KDM5C*-targeted primary AML cells.

We next assessed the clinical relevance of *KDM5C* expression in AML using the BloodPool [41] and TCGA [42] datasets. Due to marked sex-dependent differences in KDM5C levels (Fig. S7F), resulting from its escape from X-inactivation [29], we analyzed male and female data separately. Since low *KDM5C* expression was not associated with any specific AML subtype (Fig. S7G), we further investigated the prognostic value of *KDM5C* expression. In female patients, *KDM5C* levels did not impact overall survival (Fig. 6E and S8A), but when restricting the data to survival beyond six months, *KDM5C*-low patients displayed a tendency towards worse outcomes (Fig. 6F). In line with this, low *KDM5C* expression significantly predicted poor disease-free survival beyond six months (Fig. 6G, H) but did not impact on overall disease-free survival (Fig. S8B).

For male patients, stratification based on *KDM5C* expression alone did not influence neither overall nor disease-free survival (Fig. S8C–F). Instead, if we stratified based on the summed expression of *KDM5C* and the Y-linked *KDM5D*, *KDM5C/KDM5D*-low patients displayed a trend towards poor disease-free survival and had a significantly poorer overall survival (Fig. S8C–F). These findings suggest that *KDM5C* expression alone can predict outcome in females, while only the combined *KDM5C/KDM5D* levels are predictive in male patients. Moreover, *KDM5C* mutations in hematological neoplasms are more frequently found in female patients (10/327 women, 2/443 men; Fisher’s exact test *p* = 0.006, COSMIC [43]), suggesting a female bias contrasting a previous pan-cancer report [44].

Finally, we assessed whether the top- and bottom-quantiles of *KDM5C* expression in female patients were associated with specific mutations (Fig. 6I and S8G). Mutations in *PML*-*RARA*, which are associated with good prognosis [45], were significantly enriched in the *KDM5C*-high (top 25%) group and a similar trend was seen for *IDH1* mutations*. DNMT3A* and *K-RAS* mutations, associated with poor prognosis [46], tended to be more common in the *KDM5C*-low (bottom 25%) group (Fig. 6I). The low number of *CEBPA*-mutant samples (4 in total) in the cohort did not allow us to associate this genotype with *KDM5C* expression (Fig. S8G).

In conclusion, low *KDM5C* expression was associated with increased growth rates of human AML cell lines and predicts poor disease-free survival of in particular female AML patients. Additionally, high *KDM5C* expression correlates with mutations associated with good prognosis.

## Discussion

Epigenetic factors are frequently mutated in cancer and their perturbation can create a permissive environment for malignant transformation [2]. Aiming to identify novel epigenetic regulators in AML, we performed an shRNA-screen using a murine model of *CEBPA* mutant AML and identified KDM5C as a novel tumor suppressor. By shRNA-mediated knockdown of *Kdm5c/KDM5C*, we demonstrated tumor-suppressive properties in murine and human AML models, whereas we observed no advantageous effects in normal murine hematopoietic cells. Supporting a tumor suppressive function, *KDM5C* mutations have recently been reported in human hematological neoplasms, including AML [25–27]. However, the functional importance of KDM5C in hematopoietic malignancies was unknown.

Here, we found that long-term disease-free survival could be stratified based on *KDM5C*-expression, particularly in female patients. Although the regulation of *KDM5C* expression is poorly understood, its deregulation in AML is likely an indirect effect of other AML related mutations. Moreover, the overrepresentation of *KDM5C* mutations in female patients additionally supports *KDM5C* as a female-biased tumor suppressor. This sex bias is likely due to functional compensation by the Y-linked paralog, *KDM5D*, in males.

Functionally, we found that shRNA-mediated downregulation of KDM5C in Lp30 AML was associated with a de-differentiation phenotype. This was supported by an increased colony forming-activity, an increased expression of immature genes, and an immature immunophenotype following *Kdm5c-*KD.

We demonstrated that the tumor suppressive is mediated by both positively and negatively acting downstream effectors, including CBX6, TRIB3, ETV4 and FOS/JUN, where the first two also affect the differentiation status of Lp30 AML. Although the opposite impact of these effectors on leukemic aggressiveness appear mechanistically distinct, they might still be coupled. Indeed, it has been reported that ETV4 drives expression of the AP-1 transcription factor FOS Like1 (FOSL1) in ccRCC cells and that FOSL1 inhibits adipocyte differentiation by directly inhibiting *Cebpa* transcription [38, 47]. Concordantly, RNA-seq data revealed robust induction of *Fosl1* as well as slightly reduced *Cebpa* expression in response to *Kdm5c* KD (Supplementary Table 9). Although beyond the scope of this work, it is tempting to speculate that FOSL1-mediated CEBPA depletion and reduced CEBPA-FOS/JUN interaction work in concert to promote tumor aggressiveness.

Molecularly, we found that in the context of *Cebpa* mutant AML, KDM5C promoted the removal of H3K4me3 particularly at promoters of lowly expressed bivalent genes. These genes are down-regulated during myeloid differentiation but, consistent with the increase of H3K4me3 at their promoters, became up-regulated following *Kdm5c-*KD. Thus, we hypothesize that their upregulation is directly driving the de-differentiation phenotype (summarized in Fig. 6J). Meanwhile, *Kdm5c-*KD-mediated H3K4me3-increase at highly expressed genes did not affect transcription, suggesting that bivalent genes are particularly sensitive to transcription-promoting signals.

While loss of KDM5C has previously been reported to increase enhancer activity [35], we did not observe a correlation between H3K4-methylation changes and enhancer activity. However, downregulated genes were associated with a higher number of nearby enhancers, which could suggest a higher enhancer dependency compared with neutral and upregulated genes.

The recruitment of KDM5 family members and their functional redundancy is poorly understood. However, each KDM5 member has individual cancer implications [23, 48–51] and different binding patterns have been reported for KDM5B and KDM5C [52]. KDM5B has previously been reported to negatively affect leukemic stem cell maintenance specifically in MLL-rearranged AML [53]. In contrast, our data demonstrate a broader tumor suppressive function of KDM5C suggesting significant redundancy between KDM5 family members. In summary, we have uncovered KDM5C as a novel female-biased tumor suppressor in AML, which sustains leukemic differentiation via removal of H3K4me3 methylation at promoters of bivalently marked immature genes.

## Supplementary information

Supplementary Material

Supplementary Tables S4-S7

Supplementary Table S8

Supplementary Table S9

## Data Availability

All materials and relevant data described in this manuscript are freely available to any researcher to use for non-commercial purposes.
