# Supplementary material for: The histone demethylase KDM5C functions as a tumor suppressor in AML by repression of bivalently marked immature genes
Source: Leukemia. 2023 Jan 12;37(3):593–605. doi: 10.1038/s41375-023-01810-6 (PMC9991918; doi:10.1038/s41375-023-01810-6)
Supplement: Supplementary file 1 — Supplementary Material [file 41375_2023_1810_MOESM1_ESM.pdf]

Supplemental information:

The Histone Demethylase KDM5C Functions as a Tumor Suppressor in AML by Repression of Bivalently Marked Immature Genes

Mette Louise Trempenau[1-3], Mikkel Bruhn Schuster[1-3#], Sachin Pundhir[1-4#], Mafalda Araujo Pereira[1-3#], Adrija Kalvisa[1-4], Marta Tapia[1-3], Jinyu Su[1-3], Ying Ge[1-3], Bauke de Boer[1-3], Alexander Balhuizen[1-3], Frederik Otzen Bagger[1-4], Pavel Shliaha[5], Patrycja Sroczynska[2,3], Julian Walfridsson[2,3,6], Kirsten Grønbæk[2,3,7,8], Kim Theilgaard-Mönch[1-3,7,8], Ole N. Jensen[5], Kristian Helin[2,3,9,10] and Bo T. Porse [1-3, 8, *]

[1] The Finsen Laboratory, Copenhagen University Hospital - Rigshospitalet, Copenhagen, Denmark. [2] Biotech Research and Innovation Centre, University of Copenhagen, Denmark. [3] Novo Nordisk Foundation Center for Stem Cell Biology, DanStem, Faculty of Health Sciences, Faculty of Health and Medical Sciences, University of Copenhagen, Denmark. [4] The Bioinformatics Centre, Department of Biology, Faculty of Natural Sciences, University of Copenhagen, Denmark. [5] Department of Biochemistry and Molecular Biology and VILLUM Center for Bioanalytical Sciences, University of Southern Denmark (Odense), Denmark. [6] Present: Department of Medicine, Huddinge, Karolinska Institutet, Sweden. [7] Department of Hematology, Rigshospitalet, Copenhagen, Denmark. [8] Department of Clinical Medicine, Faculty of Health and Medical Sciences, University of Copenhagen, Copenhagen, Denmark. [9] Cell Biology Program and Center for Epigenetics, Memorial Sloan Kettering Cancer Center, New York, New York, USA. [10] The Institute of Cancer Research (ICR), London, UK

#Equal contribution

***Correspondence:**

Bo Porse

Finsen Laboratory, Rigshospitalet/University of Copenhagen

Content:

Supplemental Methods

10 Supplemental Tables

8 Supplemental Figures

Supplemental References

Supplemental methods

Animal experiments

Ethics:

All mouse experiments were conducted according to protocols approved by the Danish Animal Ethical Committee adhering to the three R's (refine, reduce, and replace) of animal experiments under supervision of veterinarians of the Department of Molecular Medicine, University of Copenhagen.. Mice were housed in individually ventilated cages.

In vivo shRNA screen:

The *in vivo* shRNA screen was performed using a published library(1) consisting of 849 shRNAs targeting 315 known or putative chromatin-associated factors sub-cloned from pGIPZ (Dharmacon) into pMLS (MSCV-LTRmir30-SV40-GFP)(2). Lp30 murine AML cells were transduced with library sub-pools (60-250 shRNAs, **Supplemental Table 4**), transplanted into sub-lethally irradiated recipient mice and analyzed four weeks later as previously described(3). Lp30 cells transduced with individual pools were injected in duplicate, and references were stored for later analysis. Amplified viral inserts were barcoded with di-nucleotide tags and subjected to next-generation sequencing as depicted in **Supplemental Table 5**. Raw sequence reads and frequencies for shRNA samples in the reference sample as well as in each of the output samples are indicated in **Supplemental Table 6**. shRNAs with less than 100 reads in the reference sample were omitted, and, to be able to assess shRNAs with zero reads in either of the output samples (i.e. strongly depleted shRNAs), these were given an arbitrary frequency of $5x10^{-8}$ (0.5 times the frequency corresponding to one read). The shRNAs were then ranked based on the average frequency fold change between the reference sample and the two output samples. Ranked shRNAs and their respective targets and frequencies in the reference- and output samples are shown in **Supplemental Table 7**. The genes in the shRNA-library were divided into subcategories based on Gene Ontology analysis. Genes with unknown function were categorized based on domain structure.

Competitive Bone Marrow Transplant (BMT) assays:

Recipients were transplanted with a 1:1 mix of target-shRNA and control-shRNA transduced Lp30 cells as previously described(3). The ratio of target/control shRNA Green Fluorescent Protein $(GFP)^+$ and control-shRNA Yellow Fluorescent Protein $(YFP)^+$ AML cells was analyzed by flow cytometry four weeks post-transplant.

Survival assay:

Recipients were transplanted with $2.5x10^4$ sorted shRNA-transduced $(GFP)^+$ Lp30 cells plus carrier cells and euthanized when moribund.

For the rescue assays with KD of *Etv4*, *Cbx6* or *Trib3*, Lp30 cells were double transduced with the relevant targeting or Scrambled shRNA constructs on GFP- and YFP encoding vectors. (GFP)+, (YFP)+ cells were sorted, and used for transplantation as described above.

For the rescue assay with JUN overexpression, shRNA-transduced Lp30 $(YFP)^+$ cells were expanded *in vivo* (three weeks), transduced with pMIG-empty or *JUN* overexpression vectors, and $1.5x10^5$ sorted double positive cells were transplanted into recipients.

In vivo expansion of leukemias for phenotyping assays:

To reach an equal disease load at BM harvest, 10^5 shRNA-control or $25x10^4$ *Kdm5c*-KD GFP^+ Lp30 or MLL-AF9 AML plus carrier cells were transplanted and harvested 3-4 weeks post-transplant.

Experiments were carried out according to protocols approved by the Danish Animal Ethical Committee. Mice were bred and housed locally at the Department of Experimental Medicine at the University of Copenhagen.

Plasmid constructs

Murine shRNAs cloned into pMLS(2) using EcoRI and XhoI. A non-targeting shRNA (shControl) was used as control. pMIG-*JUN*(4) (Addgene #40348) and empty pMIG-vector were used for rescue experiments. *Kdm5c*-targeting guide-RNAs (gRNAs) were designed using GT-Scan software(5) and cloned into the pL-CRISPR-SFFV-GFP(6) backbone using BsmBI.

Human *KDM5C*-shRNAs (sh*KDM5C*) were expressed from the pLKO.1-puro(7) backbone (Sigma). A non-targeting shRNA was included as control.

All shRNA and sgRNA sequences are listed in **Supplemental Table 1**

In vitro experiments

Competitive culture:

Murine c-Kit+ cells, Inv(16)/*Tet2*-/- cells or MLL-AF9 cells transduced with target-shRNA (GFP+) were mixed in a 1:1 ratio with competitive control-shRNA (YFP+) cells and seeded at 3-5x10^5 cells/ml. Cells were kept sub-confluent and counted and re-seeded every second day.

Colony-forming-unit (CFU) assay:

Transduced Lp30 cells were expanded *in vivo* as described above. 5,000 Fluorescence-Activated-Cell-Sorted (FACS)-sorted GFP+ Lp30 BM cells were plated in 1ml MethoCult M3434 (Stemcell Technologies). Colonies were counted after eight days.

Human cell lines:

For growth curves, cells were counted and reseeded at 4x10^5 (NB-4) or 2x10^5 (HL-60) cells/ml in fresh 2ug/ml puromycin supplemented medium at each time point. Doubling times were calculated using GraphPad Prism's Exponential growth equation for individual replicates (Y_0= cell number at day 0).

Cell culture conditions:

Human cell lines were acquired from ATCC and passaged before freezing. Murine cells were generated in-house. All cell lines and primary cells were mycoplasma-tested before freezing and not cultured for more than 3 months after thawing.

Lp30 cells(8) were thawed one day prior to transduction and cultured in X-vivo complete medium(3), added 10ng/mL murine GM-CSF (Prepotech) and maintained at a density of 5x10^5 cells/mL.

MLL-AF9 cells derived from a MLL-AF9-transduced murine AML(1) were cultured in R20/20 medium consisting of RPMI-1640 medium (Life Technologies) supplemented with 20% fetal bovine serum (Hyclone), 20% WEHI conditioned medium(9), 20 ng/ml SCF (Peprotech), 10 ng/ml hIL-6 (Miltenyi), and 1% penicillin/streptomycin (PAA). The cells were maintained at a density of 1.5-3x10^5 cells/mL and split every second or third day.

Inv(16)/*Tet2*-/- cells were cultured in StemSpan™ SFEM II medium (Stem Cell Technologies) containing 5mg/mL Primocin (Invitrogen), human 20ng/ml IL-6 (Peprotech), 10ng/ml murine IL-3 (Peprotech), 20ng/ml

murine GM-CSF (Peprotech), 20ng/ml murine G-CSF (Biolegend), 50ng/ml murine SCF (Peprotech), 10ng/ml murine IL-1ß (Biolegend), 50ng/mL murine FLT3L (Biolegend), 10mM Stem Reginin-1 (Stem Cell Technologies), and 10mM UM729 (Stem Cell Technologies)., 1% penicillin/streptomycin (PAA) The cells were maintained at a density of 1-3x10^5 cells/mL and split every second or third day.

c-Kit$^+$ BM: Wild-type (WT) murine BM cells were enriched for c-Kit$^+$ cells by positive selection on LS columns (Miltenyi Biotec) following incubation with anti-CD117 microbeads (Miltenyi Biotec). c-Kit-enriched cells were cultured in R20/20 medium. The cells were maintained at a density of 5x10^5 cells/mL and split every second or third day.

NB-4 cells were cultured in RPMI-1640-GlutaMAX™ (Thermo Fisher Scientific) with 10% added fetal calf serum (FCS) (HyClone) and 1% penicillin/streptomycin (PAA).

HL-60 cells were grown in DMEM with high glucose and pyruvate (Life Technologies), supplemented with 20% FCS (HyClone) and 1% penicillin/streptomycin (PAA.)

Phoenix-Eco and HEK293FT were cultured in tissue-culture treated dishes (TPP) in DMEM with high glucose and pyruvate (Life Technologies), 10% FCS (Hyclone) and 1% penicillin/streptomycin (PAA). Cells were passaged 1:5 every two days by washing with PBS and trypsinization (0.25% Trypsin-EDTA, Life Technologies).

Primary human AML cells were were cultured in StemSpan™ SFEM II medium (Stem Cell Technologies) containing 5mg/mL Primocin (Invitrogen), 20ng/ml human IL-6 (Peprotech), 10ng/ml human IL-3 (Peprotech), 10ng/ml human IL-1beta (Peprotech), 20ng/ml human GM-CSF (Peprotech), 20ng/ml human G-CSF (Biolegend), 10 ng/ml human SCF (Peprotech), 50ng/mL human FLT3L (Stem Cell Technologies), 500nM Stem Reginin-1 (MedChemExpress), and 1uM UM729 (Selleckchem), 1% penicillin/streptomycin (PAA). Cells were seeded at a density of 5 x 10^5 cells/ml, supplied with 1 volume of fresh media after 2 days and split after another 2 days. All cells were cultured at 37°C and 5% CO_2.

Virus production and transduction

Retro- and lentiviral supernatants were generated by Ca_2PO_4-mediated transfection of Phoenix-Eco or HEK293FT cells as previously described(3).

For transduction, retro- or lentiviral supernatants were spun down (2,000 ×*g*, 50 min, 32°C) on RetroNectin-coated (40ug/ml, Takara) non-tissue-culture-treated plates (Falcon). Virus was loaded and spun down twice. Cells were seeded at densities of $5x10^5$ cells/well (Lp30, c-Kit$^+$ murine cells, NB-4) or $3x10^5$ cells/well (MLL-AF9, HL-60). For Lp30 cells, the transduction was repeated the following day. All cells were expanded on the virus-coated plates for 24h prior to FACS sorting and transplantation. Cells transduced with pLKO.1-puro constructs were selected with puromycin (2ug/ml, Sigma) for three days.

MLL-AF9 CRISPR culture assay

MLL-AF9 cells were transduced with lentiviral CRISPR-constructs(6) targeting *Kdm5c* or a control. Guide-RNA sequences are listed in **Supplemental Table 1**. Bulk GFP$^+$ cells were sorted and expanded for a week. For the growth curve, cells were seeded at $1.5x10^5$ cells/ml. Cells were kept sub-confluent and counted and re-seeded every second or third day. Doubling times were calculated using GraphPad Prism's Exponential Growth Equation for individual replicates. Y_0= number of seeded cells at day 0. CRISPR-efficiency was quantified by TIDE-analysis(10) at day 0 and day 10 of the growth curve. Primers are listed in **Supplemental Table 3**.

Competitive growth assay of primary AML cells:

Cells were cultured 2 days after thawing, and electroporated with ribonucleoprotein complexes (RNPs) generated as follows: 3ug Alt-R® S.p. HiFi Cas9 (Integrated DNA technologies)was mixed with 3.2ug of sgRNA ,modified with 2'-O-Methyl at first 3bps and last 3bps, and 3'phosphorothioate bonds between first 3bps and last 2bps (Synthego), to get a molar ratio of 1:5, and incubated 10 minutes at 25°C. 5 x 10^5 cells were electroporated with either sgAAVS1 or sgKDM5C (**Supplemental Table 1**) in a 4D Nucleofector (Lonza) using the Primary cell P3 reagent (Lonza) and the DZ-100 protocol.

2 days post-electroporation (defined as day 0), the cells were mixed in a 1-1 ratio and cultured for another 16 days. Insertion and/or deletion (indel) frequencies were quantified using ICE (https://ice.synthego.com/#/) at day 0 and day 16. Primers are listed in **Supplemental Table 3**.

Frozen bone marrow aspirates from AML patients were obtained with a written informed consent in accordance with the Declaration of Helsinki under the following ethical approval from the Danish National Ethics Committe: 1705391.

Flow cytometry

Sort/competitive analysis:

Cells were washed with PBS/3%FBS and stained with 7-AAD (Invitrogen, 1:1,000) before sorting GFP$^+$ (AriaI or AriaIII, BD Biosciences) or analyzing GFP/YFP cell ratio (LSRII, BD Biosciences).

Antibody staining:

20 million Lp30 cells per sample were pelleted (300g, 5 min., 4°C), stained in 200ul antibody cocktail for 30 min, washed with 1mL PBS/3% FBS (300g, 5 min., 4°C), and resuspended in 500ul PBS/3% FBS. 7-AAD was added just before analysis on LSRII (BD Biosciences).

Immunophenotype :

Antibody cocktail: CD45.2 (PE-EF610; 1:50) CD45.1 (PECy7; 1:50), Mac1 (APC; 1:800), FcγR II/III (Alexa700; 1:100), c-Kit (Alexa780, 1:200), CD3 (PE-Cy5; 1:400), Ter119 (PE-Cy5; 1:200), and B220 (PE-Cy5; 1:400).

Apoptosis analysis:

Lp30 BM cells (20mio.) were stained in antibody cocktail (15 min. on ice) and washed with 1mL PBS/3%FCS (300g, 5 min., 4°C). Samples were re-suspended in Annexin-V-binding buffer and added 2µl Annexin-V-PE (BD Bioscience) and left to incubate at RT for 5 min in the dark. Cells were kept on ice until running on a BD LSRII. 3.5µl of (70ug/ml) DAPI solution (Invitrogen) was added to the samples just before running the samples. Single stains and fluorescence-minus-one samples (FMOs) were used to set up cytometer settings and cell gates.

Antibody cocktail: CD45.2 (PE-EF610; 1:50) CD45.1 (PECy7; 1:50), Mac1 (APC; 1:800), FcγR II/III (Alexa700; 1:100), c-Kit (Alexa780, 1:200), CD3 (PE-Cy5; 1:400), Ter119 (PE-Cy5; 1:200) and B220 (PE-Cy5; 1:400).

Cell cycle analysis:

Lp30 BM cells (20mio.) were stained in antibody cocktail (15 min on ice) and washed with 1mL PBS/3%FCS (300g, 5 min, 4°C). Cells were fixed by resuspension in 2% paraformaldehyde (Sigma) and incubated in the dark (RT for 10 min). Cells were washed with 1mL PBS/3%FCS (300g, 5 min, 4°C) and permeabilized by resuspension in 200µl Perm Buffer (BD). Incubation was performed in the dark at room temperature for 45 min, followed by a washing of the cells with 1mL PBS/3%FCS (200g, 5 min, 4°C). Cells were re-suspended in 0.5 mL of DAPI (Invitrogen) staining solution (0.5ug/mL) and incubated at room temperature for 30 min in the dark. Excess DAPI was washed away by adding 1mL of PBS/3%FCS (200g, 5 min, 4°C) before running the samples. Single stains and FMOs were used to set up cytometer settings and cell gates. The frequencies of total Lp30 cells in G1, S and G2-M-phase were determined by Dean-Jett-Fox model (FlowJo, BD). The frequencies of c-Kit^{pos} and Mac-1^{pos} Lp30 cells in G0/1, and S-G2-M-phase were determined by manual gating.

Primary antibody cocktail consists of: CD45.2 (PE-EF610; 1:50) CD45.1 (PECy7; 1:50), Mac1 (APC; 1:800), FcγR II/III (Alexa700; 1:100), c-Kit (Alexa780, 1:200), CD3 (PE-Cy5; 1:400), Ter119 (PE-Cy5; 1:200) and B220 (PE-Cy5; 1:400).

Antibody suppliers and catalog numbers are listed in **Supplemental Table 2**.

All flow cytometry data was analyzed using the FlowJo 10.1 software (BD).

Western blotting

1x10^{6} GFP positive FACS-sorted cells were washed twice with cold PBS. Cell pellets were then lysed in 100 µl of 4×Laemmli Sample buffer (Bio-Rad) with freshly added 10% β-mercaptoethanol (Sigma-Aldrich). The lysates were boiled for 5 minutes, diluted 1:2 in lysis buffer (300 mM NaCl, 50 mM 2-[4-(2-hydroxyethyl)piperazin1-yl]ethanesulfonic acid (HEPES), 1 mM EDTA, 1% Igepal CA-630 and 0.25% sodium deoxycholate) and boiled for another 5 min. 30 microliters of the resulting protein lysate was separated by SDS-PAGE on a 3-8% gel (Invitrogen).

Protein was transferred to polyvinylidene difluoride (PVDF) membranes, which were blocked with 5% skim milk in tris-buffered saline supplemented with 0.05% Tween 20 (TBST). After that, the membranes were incubated overnight at 4°C with KDM5C antibody (Abcam, ab190180, 1:1000 in TBST) and hereafter for 1 h at room temperature with HRP-conjugated polyclonal swine anti-Rabbit

immunoglobulins (Dako, P0217, 1:4000 in TBST). Proteins were detected by ECL prime western blotting system (GE Healthcare, RPN2232). Subsequently, the membranes were stripped with 0.2 M sodium hydroxide and washed with TBST, followed by incubation of monoclonal anti-β-actin-peroxidase antibody produced in mouse (Sigma-Aldrich, A3854, 1:4000 in TBST) for 1 h at room temperature. β-actin protein bands were detected as described for KDM5C.

Chromatin immuno-precipitation (ChIP)

GFP^+ Lp30 cells were FACS-sorted fresh from murine BM. Chromatin from $5x10^5$ cells per sample was crosslinked with 1% formaldehyde (Sigma), lysed and fragmented using the Diagenode Bioruptor Plus (15 cycles, 30 seconds on/30 seconds off at high setting) following the small-cell-number protocol(11) to 150-300bp. For the H3K27me3-ChIP, 25000 HEK-293 cells (fixed in 1% formaldehyde for 10 minutes) were spiked-in during the lysis step and chromatin was sonicated to 200-700bp length. Fragmented chromatin was incubated with antibodies overnight rotating at 4°C. The antibody-bound chromatin was captured with Protein-A Sepharose beads (Sigma), washed, de-cross-linked with protease K at 65°C overnight as previously described(11).

Washing procedures were optimized for the individual antibodies as follows:

H3K4me3-ChIP: 2x rinse and 2x wash in with RIPA-low (140 mM NaCl, 10 mM Tris-HCl, 1 mM EDTA, 1% Triton X-100, 0.1% SDS, 0.1% sodium deoxycholate, 1 mM PMSF), followed by 2x wash in RIPA with 0.5 M NaCl (RIPA-high), 1x wash in LiCl buffer (250 mM LiCl, 10 mM Tris-HCl, 1 mM EDTA, 0.5% Tergitol (type NP-40) and 0.1% sodium deoxycholate) and 2x wash in TE buffer (10 mM Tris-HCl, 1 mM EDTA, pH=8). H3K27me3-ChIP: 2x rinse and 3x wash in with RIPA-low, followed by 1x wash in RIPA-high, 1x wash in LiCl buffer and 2x wash in TE buffer. H3K4me1/H3K27ac/IgG-ChIP: 2x rinse in RIPA-low followed by 4x wash in RIPA-low, 1x wash in LiCl buffer and 2x wash in TE buffer. All chemicals from Sigma.

After de-crosslinking, DNA was purified using Qiagen minElute columns. DNA libraries were prepared following NEBNext® Ultra II DNA Library Kit for Illumina (New England Biolabs) guidelines and sequenced on Illumina® NextSeq500.

ChIP-seq analysis

ChIP-seq reads were mapped to mouse genome assembly (mm9) using Bowtie2(12), and converted to bigwig (duplicates removed and transcripts per million (TPM) normalized) using deepTools (bamCoverage)(13). For H3K27me3 ChIP-data, we included spikeIn (hg19) and input controls; we here made a combined genome index of mm9 and hg19 to map the raw reads using Bowtie2(12). Additionally, uniquely mapped and PCR duplicate collapsed reads mapping to mm9 and hg19 were separated, and total reads mapping to hg19 were used to scale the reads counts in mm9 as in Orlando et al. 2014(14) (scale factor = 1000000/total mapped reads in hg19). Read counts were further subtracted from corresponding counts derived from input control.

We identified a reproducible set of H3K4me1-, H3K4me3-, H3K27ac, and H3K27me3-enriched regions between biological duplicates using Irreproducible Discovery Rate (IDR)(15) (default cutoff of FDR<0.01) and defined 46,594 regions enriched for H3K4me1 and/or H3K27ac peaks in control or *Kdm5c*-KD cells. KDM5C ChIP-seq data (ESC and NPC) was retrieved from GEO (GSE34975)(16) and were processed with IDR as above. Peak regions not overlapping with promoter regions (1,000 bp region up-stream of the TSS) are annotated as enhancers (N=38,596). Normalized histone modifications and KDM5C signals (TPM) were computed for each promoter and enhancer region using a fixed-length window to the center of promoter (1,000 bp) and enhancer regions (500 bp). In case of H3K27me3, read counts were normalized using spikeIn scaling factor. Each enhancer was paired with its closest gene (Refseq; N=24,446) as its potential regulatory target. Ngs.plot.r was used to plot enrichment plots as heatmaps(17). CpG island information was obtained from UCSC table browser.

The signal strength of H3K27ac, H3K27me3, H3K4me1 and H3K4me3 in *Kdm5c*-KD vs. control within promoter and enhancer regions was quantified using BEDTools(18) (multicov -q 10 -D), and differential signals were determined using DESeq2(19) (downregulated: Log2FC<0, upregulated: Log2FC>0; FDR<0.05). The plots were visualized using ggplot2(20).

Histone mass spectrometry preparation and analysis

Kdm5c knockdown or shRNA control Lp30 cells from mouse bone marrow were sorted on a BD FACS ARIA I for GFP-expression 25 days post transplantation. Cells were washed with ice-cold PBS and snap-frozen as cell pellets. Histones were extracted from 4 million cells per replicate sample as described by

Sidoli et al(21) with optimized nuclei sedimentation at 2,000g. Trichloroacetic acid (TCA) precipitation was substituted with desalting using disposable SEC PD-10 columns (GE Healthcare, #17085101) and the samples were subsequently concentrated by SpeedVac to 100ul. The histone concentration was quantified by bicinchoninic acid (BCA) assay (each sample produced 20-30ug) and 10ug were digested with Trypsin and derivatized with phenyl isocyanate in 30ul of buffer, as previously decribed(22). Histones were desalted using 3mg of 30um Oasis Hydrophilic-Lipophilic Balance (HLB) material (Waters, cat 186007549) as recommended by the manufacturer. Desalted histone peptides were resuspended with solvent A (0.1% v/v formic acid) to a final concentration of 200ng/ul total histones. The samples were analyzed using the Data-Independent Acquisition (DIA) strategy with high resolution orbitrap MS1 and low-resolution ion trap MS2 scans(23) on Orbitrap Velos (Thermo Fisher Scientific) using nanoAcquity UPLC system (Waters), operating in direct injection mode. Peptides (600ng per injection) were directly loaded on 30cm long with 150uM inner diameter packed with intertsil 2um sorbent using flashPack method(24). The flow rate was kept at 1ul/min and column temperature was 30°C during analysis. The aqueous mobile phase A was 0.1% formic acid, the organic mobile phase B was acetonitrile with 0.1% formic acid. The gradient was 4-25% B in 49 min, 25-40% in 13 minutes, 40-80% in 4 minutes. Peptides were electro-sprayed through etched emitters according to Kelly et al(25). MS1 scans were performed at 15K resolution in Orbitrap analyzer, for MS2 peptides were isolated in ten 80Da windows and fragmented using collision induced dissociation (CID) with 35NCE. To improve peak definitions every scan was acquired with two microscans.

The results were analyzed by epiProfile(26), and the code was modified to specify phenyl isocyanate (119.037114) as N-terminal modification. The epiProfile-data are listed in **Supplemental Table 8**.

RNA-sequencing and sample preparation

RNA from 500,000 sorted GFP$^+$ Lp30 cells from each transplanted mouse was purified using the RNeasy Micro kit (Qiagen). Before purification, 5ul 100x diluted ERCC Spike-in Mix (Thermo Fischer Scientific) was added to each cell lysate.

Indexed cDNA libraries were made from 250ng of each RNA sample using the TruSeq V2 protocol (Illumina). The libraries were pooled in an equimolar ratio, PhiX Spike-in was added to 1:100 ratio, and

single-ended 75bp sequencing was performed on a NextSeq 500 (Illumina) using the High Output Flow Cell Cartridge V2.

RNA-seq data analysis

Reads were mapped to mouse genome assembly (mm9) using STAR(27), and gene expression levels were quantified using featureCounts(28) (GTF.featureType=exon, countMultiMappingReads=Y, fraction=T) on Gencode gene annotation (release M1)(29). A total of 17,815 genes with >5 reads in at least two samples were selected for differential expression analysis using the RUVSeq R-package (RUVg function)(30). Differentially expressed genes were determined separately for each of the two shRNAs (WT vs shRNA1 and WT vs shRNA2), and factors of unwanted variation between samples were determined using 568 non-differentially expressed genes (FDR>0.95 in both shRNA comparisons). Finally, significantly down- and up-regulated genes out of 12,530 protein coding genes were determined based on the criteria of logFC>0.5 or < –0.5 (in both shRNAs) and FDR<0.05 (either of the two shRNAs) (**Supplemental Table 9**). This analysis gave us a total of 167 down-regulated, 322 up-regulated and 12,041 neutral genes upon *Kdm5c*-KD that were used for further analysis.

Genes (N=12,530) were further classified as bivalent (N=451) if their promoter (±500 bp to TSS) is enriched for both H3K4me3 and H3K27me3 peaks, as repressed (N=593) if enriched for H3K27me3 peaks, as active (N=9020) if enriched for H3K4me3 peak, and remaining 2466 genes having no enrichment of either of the two histone modifications at their promoters (**Supplemental Table 9**).

RNA-seq data for normal hematopoietic populations were obtained from Pundhir et al 2018(31).

Bivalent gene set for Gene Enrichment Analysis (GSEA)

The curated bivalent gene set for GSEA was defined as the list of genes (n=854) with the presence of both H3K4me3 and H3K27me3 ChIP-seq peaks at their promoters in shRNA control Lp30 cells (**Supplemental Table 9**).

Gene Set Enrichment Analysis (GSEA)

GSEA was performed on sh*Kdm5c* (both shRNAs as one group) vs shControl Lp30 AML expression profiles using the Broad Institute Desktop software(32, 33). GSEA was based on C2 and C5 MSigDB

gene sets, a curated gene set for bivalent promoters (defined above) and curated hematopoietic signatures (based on published data(34-36), kindly provided by Claus Nerlov). For all gene sets, 1,000 permutations and Signal2Noise metric were used to assess statistical significance.

Patient data

Mutational data from patients with hematopoietic neoplasms were obtained from Catalog Of Somatic Mutations in Cancer (COSMIC)(37). Studies(38-42) with reported patient sex were used to calculate sex specific mutation frequencies.

For the BloodPool data(43) analysis, RNA expression data was downloaded from BloodSpot.eu(43). The sex of healthy donors and patients was determined by massiR(44) analysis based on the expression of Y-specific probes using the default settings (threshold=3).

For the TCGA AML data(45) analysis, RNA-seq data and clinical attributes (sex, survival data and disease-free survival data) of AML patients of all cytogenetic risk groups were downloaded from c-Bioportal(46). Patients were grouped by sex and divided based on high (above median) and low (below median) *KDM5C* expression. Male AML patients were additionally grouped based on high (above median) and low (below median) of the summed of *KDM5C* and *KDM5D* expression (only including patients where both KDM5C and KDM5D data were available). Long-term survival and long-term disease free-survival was defined by a lower cutoff of six months post diagnosis.

Supplemental tables

Supplemental Table 1: ShRNA and gRNA sequences

Murine shRNA (pGIPZ ID)	Sequence
shControl	TGCTGTTGACAGTGAGCGATCTCGCTTGGGCGAGAGTAAGTAGTGAAGCCA CAGATGTACTTACTCTCGCCCAAGCGAGAGTGCCTACTGCCTCGGA
shFos (V2LHS_42860)	TGCTGTTGACAGTGAGCGCAAGCATTGTTTGCTTATTGTTTAGTGAAGCCA CAGATGTAAACAATAAGCAAACAATGCTTATGCCTACTGCCTCGGA
shJun (V2LMM_72808)	TGCTGTTGACAGTGAGCGCCCTTTGCTTGTGCATATTTATTAGTGAAGCCAC AGATGTAATAAATATGCACAAGCAAAGGTTGCCTACTGCCTCGGA
shKdm5c-I (V2LMM_66733)	TGCTGTTGACAGTGAGCGAGCATTGTTTATCCCTATGAAATAGTGAAGCCA CAGATGTATTTCATAGGGATAAACAATGCGTGCCTACTGCCTCGGA
shKdm5c-II (V2LHS_33786)	TGCTGTTGACAGTGAGCGCGCCCAGTTTATTGAGTCATATTAGTGAAGCCA CAGATGTAATATGACTCAATAAACTGGGCATGCCTACTGCCTCGGA
shTrib3	CCGGTTGGAGTTTGATGACAATATTCTCGAGAATATTGTCATCAAACTCCA ATTTTTG
shCbx6	CCGGGCAGTCCAATTTGCTTTATTTCTCGAGAAATAAAGCAAATTGGACTG CTTTTTG
shEtv4	CCGGTGGAGGCAGGCCAAATCTAAACTCGAGTTTAGATTTGGCCTGCCTCCA TTTTTG
Murine guide-RNAs	Sequence
gControl	GCGAGGTATTCGGCTCCGCG
g1-Kdm5c	TAGGCCCAGCCCCATCATCT
g2-Kdm5c	TTTCCGCAGAGTCTTATCTT
Human shRNAs	Sequence
shControl	CCGGCAACAAGATGAAGAGCACCAACTCGAGTTGGTGCTCTTCATCTTGTTG TTTTTG
shKDM5C-I	CCGGTCGCAGAGAAATCGGGCATTTCTCGAGAAATGCCCGATTTCTCTGCGA TTTTTG
shKDM5C-II	CCGGAGTACCTGCGGTATCGGTATACTCGAGTATACCGATACCGCAGGTACT TTTTTG
Human single guide-RNAs	Sequence
sgAAVS1	GGGGCCACUAGGGACAGGAU
sgKDM5C	CUUACCGCCAUGACACACUU

Supplemental Table 2: Antibodies

Antibody	Supplier	Product number
B220 PE-Cy5	eBioscience	15-0452-83
CD11b (Mac-1) APC	BioLegend	101210
CD16/32 A700	eBioscience	56-0161-82
CD3e PE-Cy5	eBioscience	15-0031-82
CD45.1 PE-Cy7	BD	560578
CD45.2 PE-EF610	eBioscience	61-0454-82
c-Kit A780	eBioscience	47-1171-82
Ter119 PE-Cy5	BioLegend	116210

Supplemental Table 3: Primer sequences

Murine RT-qPCR primers for cDNA expression	Forward	Reverse
Actb	GGAGGGGGTTGAGGTGGT	TGTGCACTTTTATTGGTCTCAAC
Fos	TCCGGTTCCTTCTATGCAGC	GTGACCACGGGAGTACACAG
Jun	CAAAACTCCGAGCTGGCATC	GCGTTAGCATGAGTTGGCAC
Kdm5a	AGGTGCCATGGCTCTATGTG	CTCGCCCCAATGCAAGTAGT
Kdm5b	GCACTAGGACTGGCAAGGTAG	TGCCAATCCTCGGAAAGTACG
Kdm5c	TTGGAGAGATGGCTGACTCC	TTCCTCGATGCTATTCACCA
Etv4	GGACCTCAGTCACTTCCAAGA	GAACAAACTGCTCATCACTGTCC
Cbx6	TGTATGGGCCCAAGAAGAGG	ATCACTGATGCGGAGGGC
Trib3	AGCTATCGAGCCCTGCACT	AGCCGGGCATAAGGTGC
Human RT-qPCR primers for cDNA expression	Forward	Reverse
KDM5C	TTGTCTGCCTTTCCCACATC	AGCCCGAACCTTCAGCTTAT
POLR2A	TCTTCTGCTCCAAACTGCTTGTG	ATGTGTGAGCCGCTTCTTGG
Murine PCR primers for TIDE-analysis	Forward	Reverse
g1-*Kdm5c*	GAGTTGATTCTTCCCTTCTACCACC	GTTCCTTCGAAGCCTCATGGT
g2-*Kdm5c*	TGTCAGGCTTGATGACTTAGCC	CTTCGAAGCCTCATGGTCATCT
Human PCR primers for ICE-analysis	Forward	Reverse
sg*KDM5C*	GCTGCTTTGAGTGCTTCCTT	CTCCTGCTCCTTTCTCCCAA
sg*AAVS1*	AGGGAGTTTTCCACACGGAC	CAAAGTACCCCGTCTCCCTG

Supplemental Table 4: ShRNA pools for screen

Included as additional file.

Supplemental Table 5: Di-nucleotide tags for sequencing of shRNA pools

Included as additional file.

Supplemental Table 6: Read counts and individual shRNA frequencies

Included as additional file.

Supplemental Table 7: Ranked list with shRNA foldchanges

Included as additional file.

Supplemental Table 8: Histone PTM frequencies for individual peptides

Included as additional file.

Supplemental Table 9: RNA-seq expression matrix

Included as additional file.

Supplemental Table 10: KDM5C mutations in hematopoietic malignancies

Mutation type	AA mutation	Genomic position (GRCh38)	Patient sex	Hematopoietic malignancy	pubmed ID	Cosmic ID
Missense	p.E208G	X:53215934	Female	Adult T cell lymphoma-leukaemia	26437031	COSM5711701
Missense	p.E275G	X:53215934	Female	Adult T cell lymphoma-leukaemia	26437031	COSM5711701
Missense	p.R1481G	X:53193209	Female	Adult T cell lymphoma-leukaemia	26437031	COSM5711699
Missense	p.G1067E	X:53194968	Female	Acute lymphoblastic leukaemia	COSU533**	COSM5005309
Missense	p.R677W	X:53198990	Female	Acute myeloid leukaemia	29253671	COSM7003650
Missense	p.M504V	X:53210449	Female	Acute myeloid leukaemia	26438511	COSM5985485
Substitution (intronic)	-	X:53198759	Female	Acute myeloid leukaemia	COSU544**	COSM7219030
Missense	p.N795I	X:53197808	Female	Diffuse large B cell lymphoma	26647218	COSM5652245
Missense*	p.C278R	X:53214778	Female	Diffuse large B cell lymphoma	28481359	COSM6987468
Missense*	p.D270G	X:53214801	Female	Diffuse large B cell lymphoma	28481359	COSM6987472
Missense	p.P153L	X:53216196	Male	Diffuse large B cell lymphoma	28481359	COSM6937436
Substitution (intronic)	-	X:53201852	Female	Diffuse large B cell lymphoma	28481359	COSM6987637
Missense	p.R614L	X:53201569	Male	Myelodysplastic syndrome	24850867	COSM4169836
Misssense	p.P1046S	X:53195395	NA	Diffuse large B cell lymphoma	24531327	COSM5621088
Misssense	p.Q560K	X:53210482	NA	Essential thrombocythaemia	24325359	COSM3719986
Nonsense	p.C1247	NA	NA	Myelofibrosis	24150215	COSM5881445
Misssense	p.R1059L	X:53195355	NA	Chronic lymphocytic leukaemia	23415222	COSM1293026

* Mutations found in the same patient. ** designate COSMIC study ID of unpublished studies.

Supplemental figures

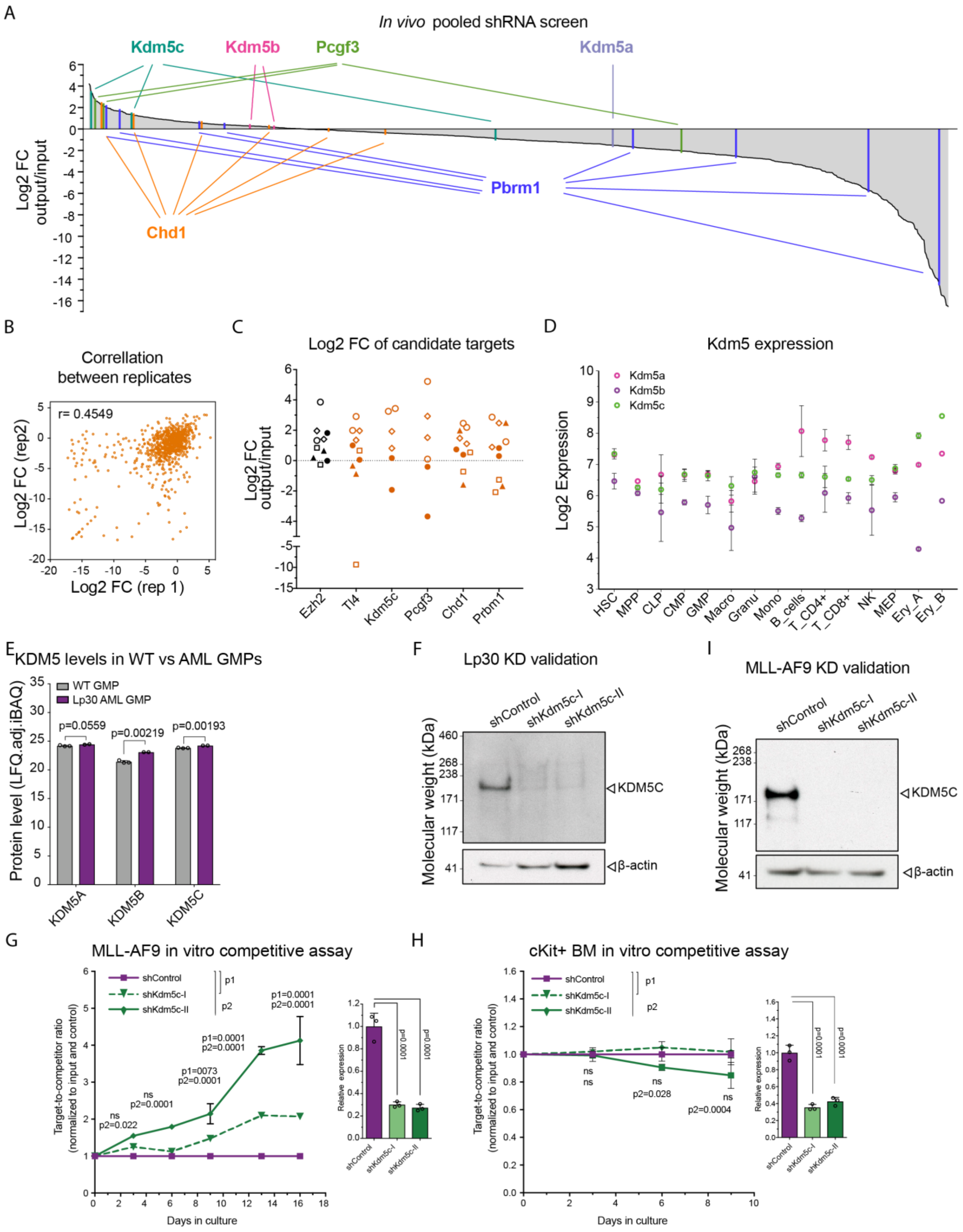

Figure S1. *In vivo* shRNA screen identifies KDM5C as a putative tumor suppressor in AML. (A) Waterfall plot of the screening results. The mean Log2 fold-change (output/input) is ranked positive (enriched) to negative (depleted). The performance of shRNAs targeting candidate genes and KDM5-family members is indicated. (B) Scatterplot depicting Spearman's correlation coefficient between the Log2 fold-change of individual shRNAs in duplicate mice. (C) Performance of selected shRNAs in biological duplicate experiments indicated by identical symbols. Different symbol shapes represent different shRNAs targeting the same gene. (D) mRNA expression of *Kdm5a* (pink), *Kdm5b* (purple) and *Kdm5c* (green) in normal murine hematopoiesis (RNA-seq, extracted from BloodSpot.eu(43)). (E) KDM5 protein levels in murine wild type (WT) and Lp30 AML cells. Based on label-free quantification (LFQ) mass spectrometry data adjusted using intensity-based absolute quantification (iBAQ). The data was obtained from Jakobsen et al 2019(47). (F) Western blot showing KDM5C protein level in shControl and *Kdm5c*-KD in Lp30 cells. (G-H) Competitive culture of control and *Kdm5c*-KD MLL-AF9 cells (G) and murine c-Kit-enriched BM cells (H) (biological triplicates). Inserts: Relative expression of Kdm5c. (I) Western blot showing KDM5C protein level in shControl and *Kdm5c*-KD in MLL-AF9 cells.

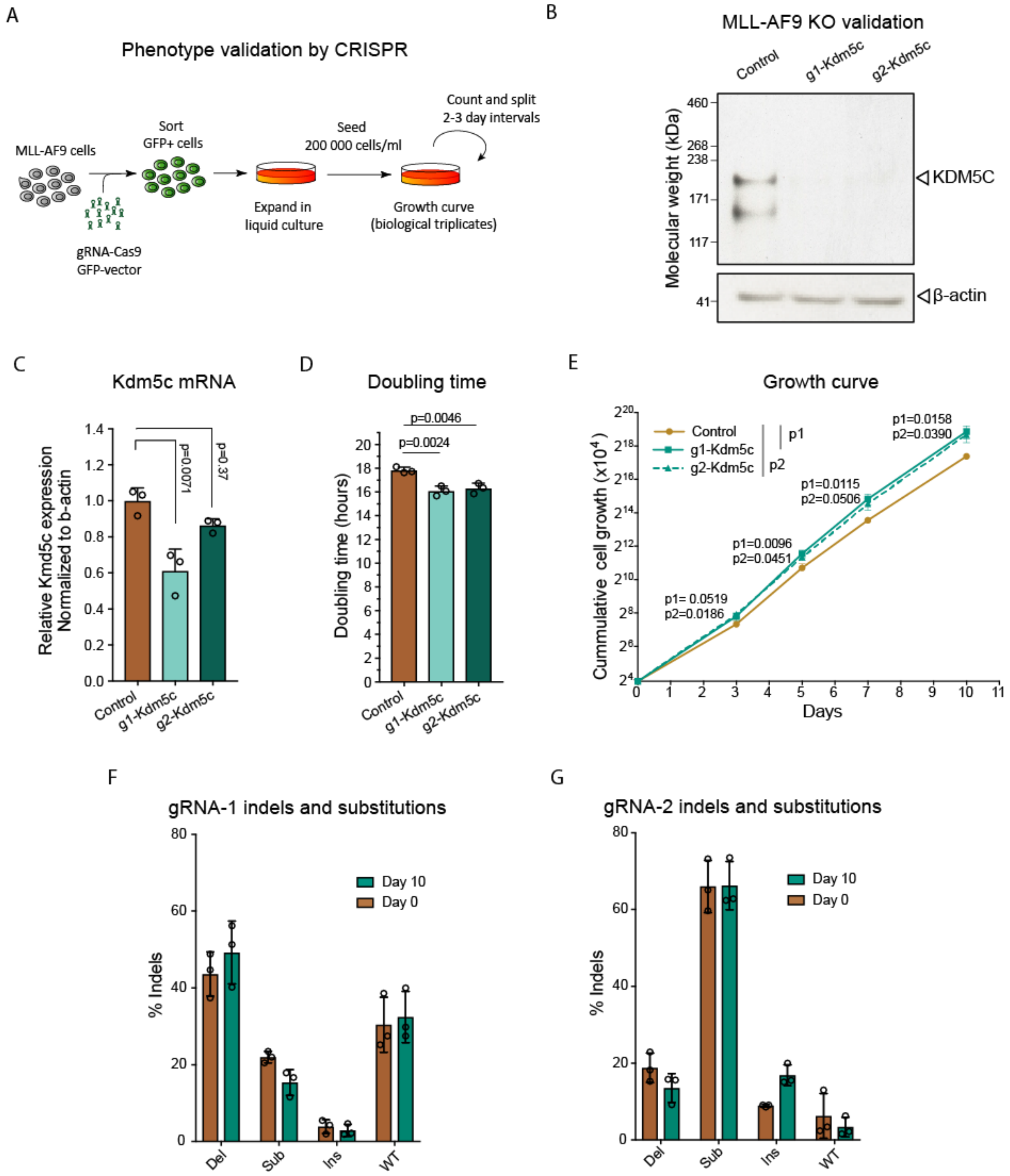

Figure S2. Validation of *Kdm5c*-KD phenotype by CRISPR-knockout in MLL-AF9 cell line. (A) Schematic outline of a CRISPR-knockout cell culture assay. MLL-AF9 cells were transduced with control or *Kdm5c*-targeting guide-RNA (gRNA) expressing lentivirus sorted for GFP-expression and expanded in culture for a week in biological triplicates. The cells were counted and passaged every 2-3 days. (B) Western blot showing KDM5C protein levels in control and *Kdm5c*-KO MLL-AF9 cells. Beta-actin level is used as loading control. (C) Relative *Kdm5c* expression in control and *Kdm5c*-KO MLL-AF9 cells assayed by RT-qPCR and normalized to *Actb* expression. (D) Mean doubling time of control and *Kdm5c*-KO MLL-AF9 cells of biological triplicates. (E) Growth curve of control and *Kdm5c*-KO MLL-AF9 cells. (F-G) Efficiency of gRNA-1 (F) and gRNA-2 (G) determined by the frequency of indels and substitutions in the *Kdm5c* gene over time (day 0 vs day 10 of growth) in the biological replicates (rep1-3). Del=deletion, Sub=substitution, Ins=insertion, WT=wild type sequence.

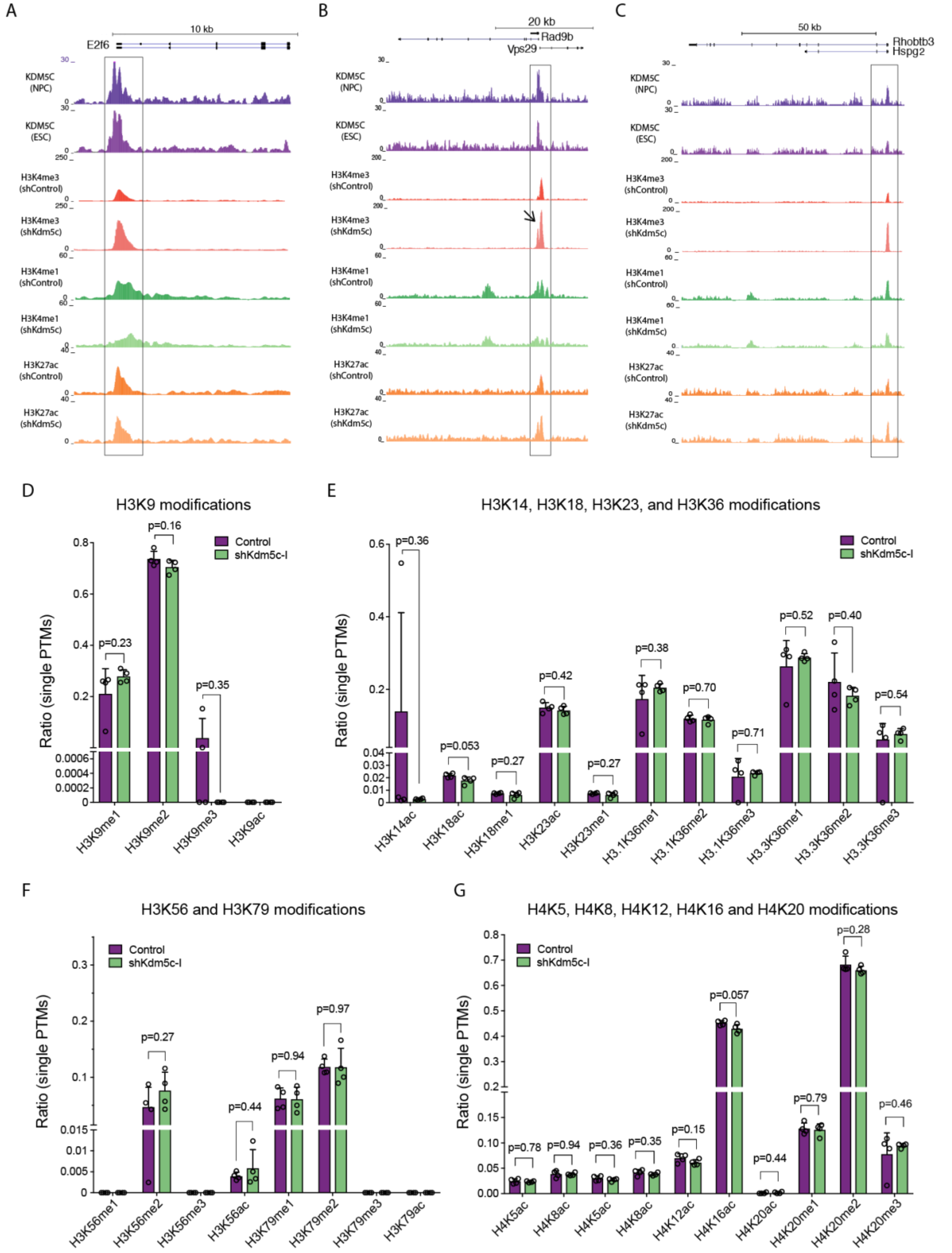

Figure S3. H3K4me3 increases in *Kdm5c*-KD cells at expected KDM5C-bound regions. (A-C) Examples of KDM5C occupancy (ESC/NPC) at promoter regions correlating with an H3K4me3-increase in *Kdm5c*-KD Lp30 cells. Boxes and arrow indicate regional changes. (D-G) Ratios of global changes in histone modifications in *Kdm5c*-KD vs control Lp30 cells: (D) H3K9; (C) H3K14, H3K18, H3K23 and H3K36; (D) H3K56 and H3K79; (E) H4K5, H4K8, H4K16 and H4K20. P-values were calculated using two-tailed T-tests (not adjusted for multiple testing).

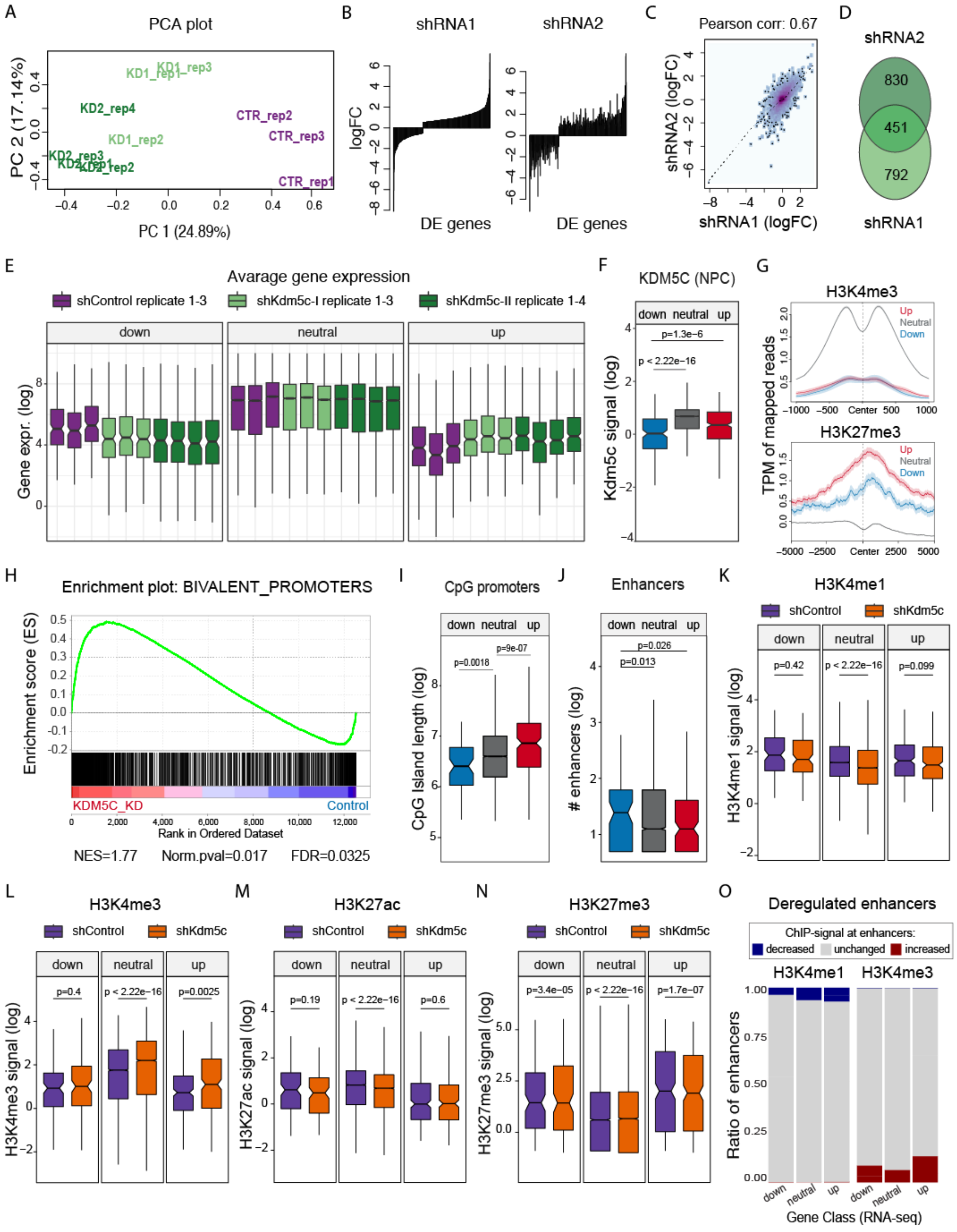

Figure S4. Deregulated genes display different promoter features. (A) Principle component analysis (PCA) of RNA-seq of replicate samples of control (CTR) and *Kdm5c*-KD (KD1; KD2) groups after normalization. (B) Fold-changes of *Kdm5c*-KD vs control. Fold-changes of deregulated genes were ranked by the shKdm5c-I (shRNA-1) group and show a similar trend of deregulated genes in the shKdm5c-II (shRNA-2) group. (C) Scatterplot depicting Pearson correlation coefficient between gene expression LogFC in *Kdm5c*-KD groups. (D) Overlap between deregulated genes in *Kdm5c*-KD groups. Cutoff: <0.05 FDR and >0.5 absolute logFC in individual shRNA-groups. (E) Notched boxplots showing average Log mRNA expression of the up, down or neutrally regulated genes in individual samples. (F) Notched boxplot showing KDM5C signal based on NPC data (Outchkourov et al 2013)(16) at promoters of up-, down- or neutrally regulated genes in *Kdm5c*-KD cells. (G) Mean H3K4me3- and H3K27me3 signals as transcripts per million (TPM) mapped reads in shControl Lp30 cells at promoters of up-, down-, or neutrally regulated genes in shKdm5c-I vs. shControl Lp30 cells. Lighter shaded ribbons represent standard error of mean. (H) Gene Set Enrichment Analysis showing enriched gene expression from bivalently marked promoters in *Kdm5c*-KD vs shControl (Lp30 cells). (I) Notched boxplots showing the lengths of CpG island at promoters as in (F). (J) Notched boxplots showing the number of enhancers near promoters of up-, down- or neutrally regulated genes in *Kdm5c*-KD cells. Outliers not shown. (K-N) Notched boxplots showing H3K4me1- (K), H3K4me3- (L), H3K27ac-signal (M), or H3K27me3 (N) at enhancer categories defined in (J). Outliers not shown. (O) Distribution of enhancers with deregulated H3K4me3 and H3K4me1 peaks. Frequency of enhancers with peak increase (red), decrease (blue) or no change (grey) closest to promoters of the gene classes (up-, down- or neutrally regulated genes in *Kdm5c*-KD cells).

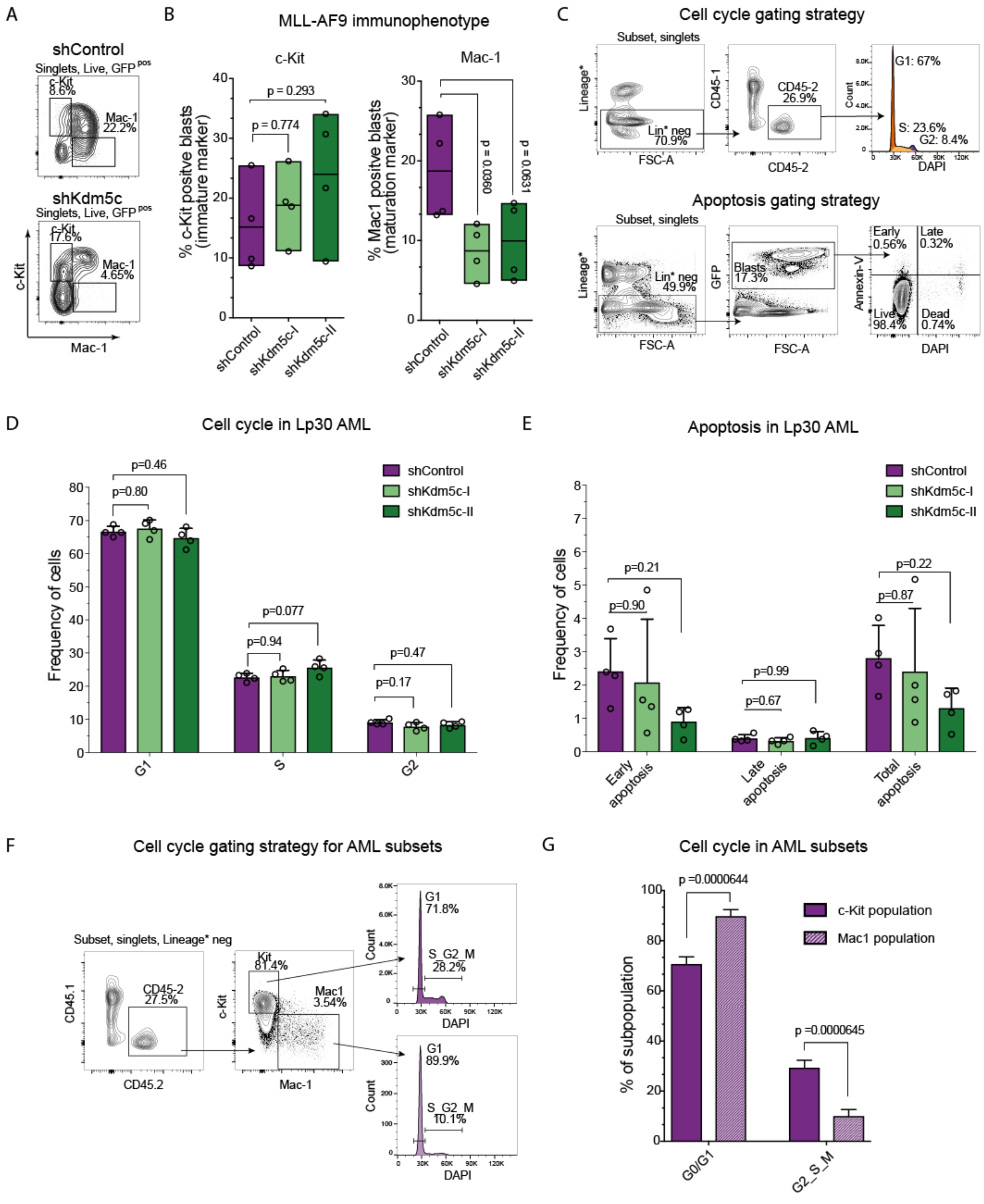

Figure S5. The *Kdm5c*-KD phenotype is not linked to changes in cell cycle or apoptosis. (A) Gating strategy for MLL-AF9 AML (singlets, DAPI negative, GFP positive, assessed in BM, 3 weeks post-BMT). (B) Frequency of control and *Kdm5c*-KD MLL-AF9 cells with immature (c-Kit+) and mature (Mac-1+) immune-phenotype *in vivo*. (C) Top panel: Gating strategy for the cell cycle assay on CD45.2^{+} Lp30 BM cells. Bottom panel: Gating strategy for the apoptosis assay on GFP^{+} Lp30 BM cells. (D) Cell cycle: Frequency of Lp30 cells in G1, S and G2M phases determined by the Dean-Jett-Fox model (FlowJo). (E) Frequency of Lp30 cells in early (Annexin-V^{+}, DAPI^{-}) and late (Annexin-V^{+}, DAPI^{+}) apoptosis. Total apoptosis is the sum of both. (F) Left panel: Flow cytometry gating strategy for the cell cycle assay on c-Kit and Mac-1 subpopulations of CD45.2^{+} Lp30 BM cells. Right panel: Cell cycle: Frequency of c-Kit^{+} and Mac-1^{+} Lp30 cells in G0/G1 and S-G2-M phases determined by DAPI staining.

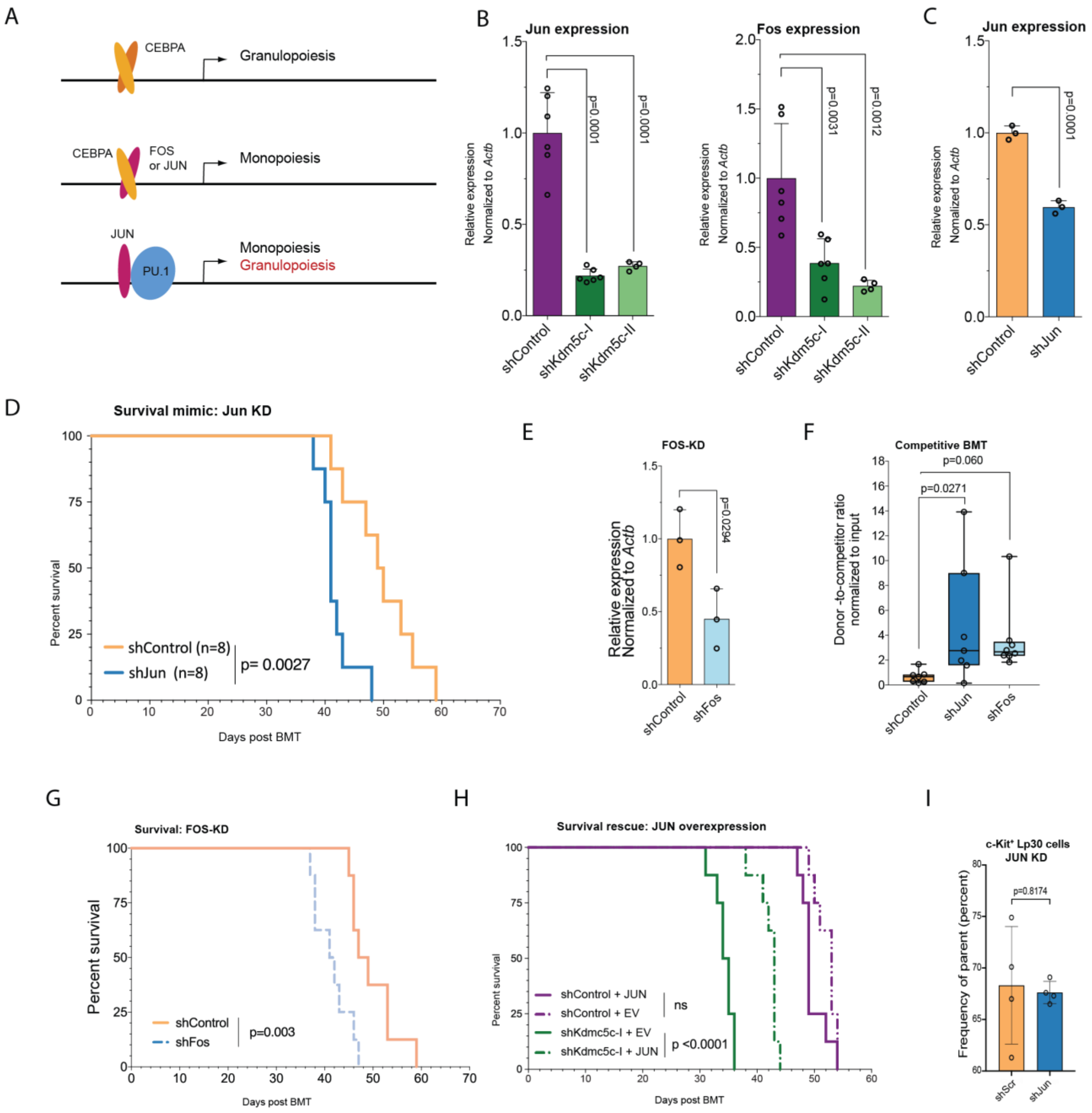

Figure S6. Direct and indirect downstream mediators facilitate the *Kdm5c*-KD phenotype. (A) Schematic model of the involvement of AP-1 factors in myeloid differentiation. AP-1 factors can dimerize with CEBPA favoring monopoiesis over granulopoiesis which is promoted by CEBPA homodimers. JUN can also act as a co-activator of PU-1 to upregulate monocytic genes. (B) RT-qPCR showing decreased *Fos* and *Jun* expressions in *Kdm5c*-KD Lp30 cells *in vivo* normalized to *Actb*-expression. (C) Relative mRNA expression of *Jun* in control and *Jun*-KD groups assayed by RT-qPCR and normalized to *Actb* expression. (D) Mimicking *Kdm5c KD*: Kaplan-Meier survival analysis of mice transplanted with control or *Jun*-knockdown Lp30 cells (n=8 per group). (E) Relative mRNA expression of *Fos* in control and *Fos*-knockdown groups assayed by RT-qPCR and normalized to *Actb* expression. (F) Performance of Lp30 *Fos* and *Jun*-KD cells in competitive BMT normalized to input ratio of target/competitor cells (n=4). (G) Mimicking *Kdm5c KD*: Kaplan-Meier survival analysis of mice with transplanted control or *Fos*-knockdown Lp30 cells (n=8 per group). (H) Rescuing *Kdm5c KD*: Kaplan-Meier survival analysis of mice transplanted with Lp30 cells transduced with either control or *Kdm5c*-shRNA in combination with empty vector (EV) or JUN-overexpression (n=8 per group) (I) Frequency of Lp30 cells with immature (c-Kit+) immunophenotype *in vivo* after transduction with either shControl or sh*Jun*.

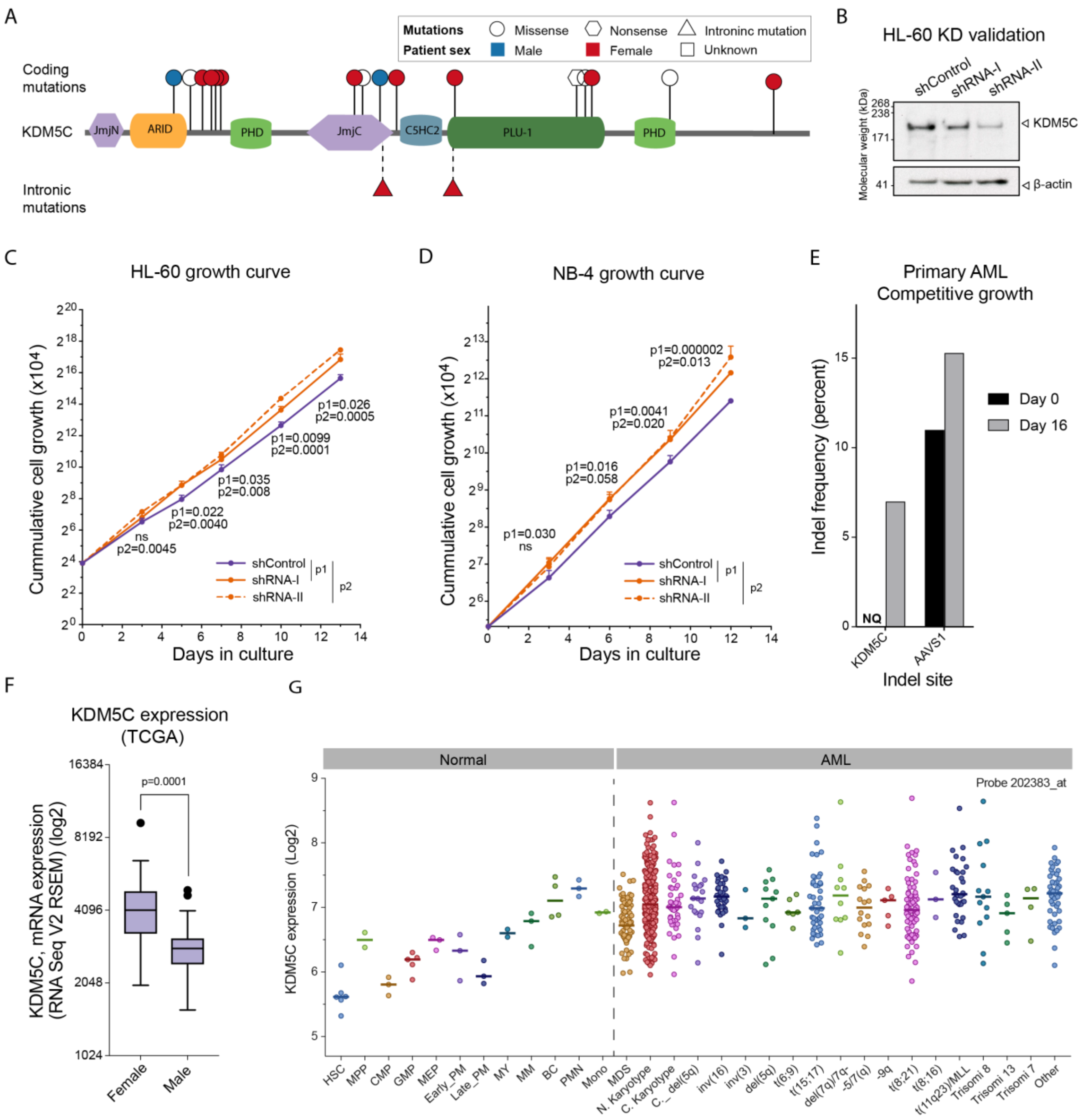

Figure S7. *KDM5C* in human AML cell lines and patients. (A) Schematic overview of the location and type (missense, nonsense or intronic) of *KDM5C* mutations in human hematopoietic neoplasms (COSMIC(37), Supplemental table 10. Red= female patient, blue= male patients, clear= patient sex unknown. (B) Western blot showing KDM5C protein level in shControl and *Kdm5c*-KD HL-60 cells. Beta-actin level is used as loading control. (C-D) Growth curve of HL-60 (C) and NB-4 (D) shControl and *KDM5C*-KD cells. P-values were calculated using multiple unpaired two-tailed T-tests. (D) Quantification of insertion and/or deletion (indel) frequencies in primary AML cells electroporated with sgKDM5C and sgAAVS1 and subjected to competitive growth. NQ: Non-quantifiable (F) Log2 mRNA expression of *KDM5C* in male and female AML patients (TCGA dataset(45)). P-value was calculated by unpaired two-tailed T-test with Welch's correction. (G) Log2 *KDM5C* mRNA expression in normal hematopoietic cells and in AML subtypes (female donors/patients from the BloodPool dataset(43)). HSC= hematopoietic stem cell, MPP= multipotent progenitor, CMP= common myeloid progenitor, GMP=granulocyte monocyte progenitor, MEP= megakaryocyte-erythroid progenitor, Early-PM= early promyelocyte, Late-PM= late promyelocyte, BC= Band cell, MM= meta myelocyte, Mono= monocyte, MY= meylocyte, PMN= Polymorphnuclear cell, MDS= myelodysplastic syndrome, N.Karyotype= AML with normal karyotype, C.Karyotype= AML with complex karyotype, C._del(q5)= AML with complex karyotype and del(q5). Additional AML subtypes according to the indicated aberrations.

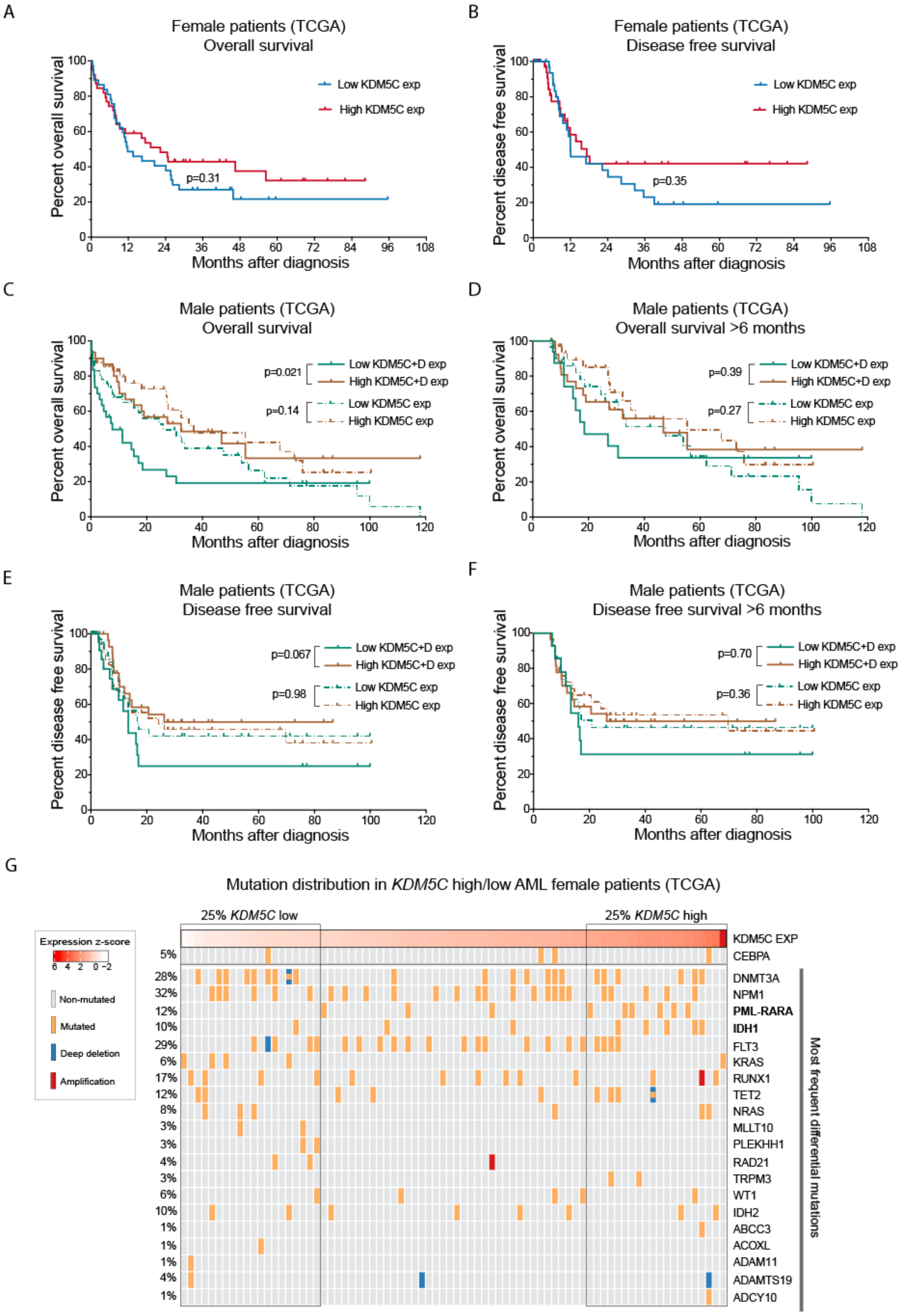

Figure S8. *KDM5C* in AML patients. (A-B) Overall survival (A) and disease-free survival (B) of female AML patients (TCGA dataset(45)) grouped according to *KDM5C* High (above median) and Low (below median) expression as indicated. (C-D) Overall survival (C) and long-term (>6 months) overall survival (D) of male AML patients (TCGA dataset(45)) grouped according to *KDM5C* or *KDM5C+D* High (above median) and Low (below median) expression as indicated. (E-F) Disease-free survival (E) and long-term (>6 months) disease-free survival (F) of male AML patients grouped as in C. (G) Distribution of *CEBPA* mutations and the 20 most frequent differential mutations in female AML patients (TCGA(45)) sorted from low to high *KDM5C* expression. Significant differentially mutated genes are marked in bold (calculated between patients in the top and bottom quantile of *KDM5C* expression). Percentages relate to the general frequency of the mutation across the female AML patients.

Supplemental references

1. Sroczynska P, Cruickshank VA, Bukowski J-P, Miyagi S, Bagger FO, Walfridsson J, et al. shRNA screening identifies JMJD1C as being required for leukemia maintenance. Blood. 2014.

2. Dickins RA, Hemann MT, Zilfou JT, Simpson DR, Ibarra I, Hannon GJ, et al. Probing tumor phenotypes using stable and regulated synthetic microRNA precursors. Nature genetics. 2005;37(11):1289-95.

3. Ge Y, Schuster MB, Pundhir S, Rapin N, Bagger FO, Sidiropoulos N, et al. The splicing factor RBM25 controls MYC activity in acute myeloid leukemia. Nature Communications. 2019;10(1):172-.

4. Wang Z-Y, Sato H, Kusam S, Sehra S, Toney LM, Dent AL. Regulation of IL-10 Gene Expression in Th2 Cells by Jun Proteins. The Journal of Immunology. 2005;174(4):2098-105.

5. O'Brien A, Bailey TL. GT-Scan: identifying unique genomic targets. Bioinformatics. 2014;30(18):2673-5.

6. Heckl D, Kowalczyk MS, Yudovich D, Belizaire R, Puram RV, McConkey ME, et al. Generation of mouse models of myeloid malignancy with combinatorial genetic lesions using CRISPR-Cas9 genome editing. Nature biotechnology. 2014;32(9):941-6.

7. Stewart SA, Dykxhoorn DM, Palliser D, Mizuno H, Yu EY, An DS, et al. Lentivirus-delivered stable gene silencing by RNAi in primary cells. RNA. 2003;9(4):493-501.

8. Kirstetter P, Schuster MB, Bereshchenko O, Moore S, Dvinge H, Kurz E, et al. Modeling of C/EBPalpha mutant acute myeloid leukemia reveals a common expression signature of committed myeloid leukemia-initiating cells. Cancer cell. 2008;13(4):299-310.

9. Lee JC, Hapel AJ, Ihle JN. Constitutive production of a unique lymphokine (IL 3) by the WEHI-3 cell line. Journal of immunology (Baltimore, Md : 1950). 1982;128(6):2393-8.

10. Brinkman EK, Chen T, Amendola M, van Steensel B. Easy quantitative assessment of genome editing by sequence trace decomposition. Nucleic Acids Research. 2014;42(22):e168-e.

11. Jakobsen JS, Bagger FO, Hasemann MS, Schuster MB, Frank A-K, Waage J, et al. Amplification of pico-scale DNA mediated by bacterial carrier DNA for small-cell-number transcription factor ChIP-seq. BMC Genomics. 2015;16(1):46-.

12. Langmead B, Salzberg SL. Fast gapped-read alignment with Bowtie 2. Nature Methods. 2012;9:357-9.

13. Ramírez F, Dündar F, Diehl S, Grüning BA, Manke T. DeepTools: A flexible platform for exploring deep-sequencing data. Nucleic Acids Research. 2014.

14. Orlando David A, Chen Mei W, Brown Victoria E, Solanki S, Choi Yoon J, Olson Eric R, et al. Quantitative ChIP-Seq Normalization Reveals Global Modulation of the Epigenome. Cell Reports. 2014;9(3):1163-70.

15. Li Q, Brown JB, Huang H, Bickel PJ. Measuring reproducibility of high-throughput experiments. Annals of Applied Statistics. 2011;5(3):1752-79.

16. Outchkourov NS, Muiño JM, Kaufmann K, van Ijcken WFJ, Groot Koerkamp MJ, van Leenen D, et al. Balancing of histone H3K4 methylation states by the Kdm5c/SMCX histone demethylase modulates promoter and enhancer function. Cell reports. 2013;3(4):1071-9.

17. Shen L, Shao N, Liu X, Nestler E. ngs.plot: Quick mining and visualization of next-generation sequencing data by integrating genomic databases. BMC genomics. 2014;15(1):284-.

18. Quinlan AR, Hall IM. BEDTools: A flexible suite of utilities for comparing genomic features. Bioinformatics. 2010;26(6):841-2.

19. Love MI, Huber W, Anders S. Moderated estimation of fold change and dispersion for RNA-seq data with DESeq2. Genome Biology. 2014;15(12):550-.

20. Wickham H. Introduction. In: ggplot2. Use R. Springer, New York, NY. 2009:1-7.

21. Sidoli S, Bhanu NV, Karch KR, Wang X, Garcia BA. Complete Workflow for Analysis of Histone Post-translational Modifications Using Bottom-up Mass Spectrometry: From Histone Extraction to Data Analysis. Journal of visualized experiments : JoVE. 2016(111).

22. Maile TM, Izrael-Tomasevic A, Cheung T, Guler GD, Tindell C, Masselot A, et al. Mass spectrometric quantification of histone post-translational modifications by a hybrid chemical labeling method. Molecular & cellular proteomics : MCP. 2015;14(4):1148-58.

23. Sidoli S, Simithy J, Karch KR, Kulej K, Garcia BA. Low Resolution Data-Independent Acquisition in an LTQ-Orbitrap Allows for Simplified and Fully Untargeted Analysis of Histone Modifications. Analytical chemistry. 2015;87(22):11448-54.

24. Kovalchuk SI, Jensen ON, Rogowska-Wrzesinska A. FlashPack: Fast and Simple Preparation of Ultrahigh-performance Capillary Columns for LC-MS. Molecular & cellular proteomics : MCP. 2019;18(2):383-90.

25. Kelly RT, Page JS, Luo Q, Moore RJ, Orton DJ, Tang K, et al. Chemically Etched Open Tubular and Monolithic Emitters for Nanoelectrospray Ionization Mass Spectrometry. Analytical Chemistry. 2006;78(22):7796-801.

26. Yuan Z-F, Lin S, Molden RC, Cao X-J, Bhanu NV, Wang X, et al. EpiProfile Quantifies Histone Peptides With Modifications by Extracting Retention Time and Intensity in High-resolution Mass Spectra. Molecular & cellular proteomics : MCP. 2015;14(6):1696-707.

27. Dobin A, Davis CA, Schlesinger F, Drenkow J, Zaleski C, Jha S, et al. STAR: Ultrafast universal RNA-seq aligner. Bioinformatics. 2013.

28. Liao Y, Smyth GK, Shi W. FeatureCounts: An efficient general purpose program for assigning sequence reads to genomic features. Bioinformatics. 2014.

29. Frankish A, Diekhans M, Ferreira AM, Johnson R, Jungreis I, Loveland J, et al. GENCODE reference annotation for the human and mouse genomes. Nucleic Acids Research. 2019.

30. Risso D, Ngai J, Speed TP, Dudoit S. Normalization of RNA-seq data using factor analysis of control genes or samples. Nature Biotechnology. 2014.

31. Pundhir S, Bratt Lauridsen FK, Schuster MB, Jakobsen JS, Ge Y, Schoof EM, et al. Enhancer and Transcription Factor Dynamics during Myeloid Differentiation Reveal an Early Differentiation Block in Cebpa null Progenitors. Cell Reports. 2018;23(9):2744-57.

32. Mootha VK, Lindgren CM, Eriksson K-F, Subramanian A, Sihag S, Lehar J, et al. PGC-1α-responsive genes involved in oxidative phosphorylation are coordinately downregulated in human diabetes. Nature Genetics. 2003;34(3):267-73.

33. Subramanian A, Tamayo P, Mootha VK, Mukherjee S, Ebert BL, Gillette MA, et al. Gene set enrichment analysis: A knowledge-based approach for interpreting genome-wide expression profiles. Proceedings of the National Academy of Sciences. 2005;102(43):15545-50.

34. Pronk CJH, Rossi DJ, Månsson R, Attema JL, Norddahl GL, Chan CKF, et al. Elucidation of the phenotypic, functional, and molecular topography of a myeloerythroid progenitor cell hierarchy. Cell stem cell. 2007;1(4):428-42.

35. Venezia TA, Merchant AA, Ramos CA, Whitehouse NL, Young AS, Shaw CA, et al. Molecular Signatures of Proliferation and Quiescence in Hematopoietic Stem Cells. PLoS Biology. 2004;2(10):e301-e.

36. Månsson R, Hultquist A, Luc S, Yang L, Anderson K, Kharazi S, et al. Molecular Evidence for Hierarchical Transcriptional Lineage Priming in Fetal and Adult Stem Cells and Multipotent Progenitors. Immunity. 2007;26(4):407-19.

37. Tate JG, Bamford S, Jubb HC, Sondka Z, Beare DM, Bindal N, et al. COSMIC: the Catalogue Of Somatic Mutations In Cancer. Nucleic acids research. 2018;47(D1):D941-D7.

38. Kataoka K, Nagata Y, Kitanaka A, Shiraishi Y, Shimamura T, Yasunaga J-i, et al. Integrated molecular analysis of adult T cell leukemia/lymphoma. Nature Genetics. 2015;47(11):1304-15.

39. Zhan D, Zhang Y, Xiao P, Zheng X, Ruan M, Zhang J, et al. Whole exome sequencing identifies novel mutations of epigenetic regulators in chemorefractory pediatric acute myeloid leukemia. Leukemia Research. 2018;65:20-4.

40. Garg M, Nagata Y, Kanojia D, Mayakonda A, Yoshida K, Haridas Keloth S, et al. Profiling of somatic mutations in acute myeloid leukemia with FLT3-ITD at diagnosis and relapse. Blood. 2015;126(22):2491-501.

41. Morin RD, Assouline S, Alcaide M, Mohajeri A, Johnston RL, Chong L, et al. Genetic Landscapes of Relapsed and Refractory Diffuse Large B-Cell Lymphomas. Clinical Cancer Research. 2016;22(9):2290.

42. Zehir A, Benayed R, Shah RH, Syed A, Middha S, Kim HR, et al. Mutational landscape of metastatic cancer revealed from prospective clinical sequencing of 10,000 patients. Nature medicine. 2017;23(6):703-13.

43. Bagger FO, Sasivarevic D, Sohi SH, Laursen LG, Pundhir S, Sønderby CK, et al. BloodSpot: A database of gene expression profiles and transcriptional programs for healthy and malignant haematopoiesis. Nucleic Acids Research. 2016;44(D1):D917-D24.

44. Buckberry S, Bent SJ, Bianco-Miotto T, Roberts CT. massiR : a method for predicting the sex of samples in gene expression microarray datasets. Bioinformatics. 2014;30(14):2084-5.

45. Ley TJ, Miller C, Ding L, Raphael BJ, Mungall AJ, Robertson G, et al. Genomic and epigenomic landscapes of adult de novo acute myeloid leukemia. New England Journal of Medicine. 2013;368(22):2059-74.

46. Gao J, Aksoy BA, Dogrusoz U, Dresdner G, Gross B, Sumer SO, et al. Integrative Analysis of Complex Cancer Genomics and Clinical Profiles Using the cBioPortal. Science Signaling. 2013;6(269):pl1-pl.

47. Jakobsen JS, Laursen LG, Schuster MB, Pundhir S, Schoof E, Ge Y, et al. Mutant CEBPA directly drives the expression of the targetable tumor-promoting factor CD73 in AML. Science Advances. 2019;4(July).
